# Supplementary material for: NF45/NF90‐mediated rDNA transcription provides a novel target for immunosuppressant development
Source: EMBO Mol Med. 2021 Feb 8;13(3):e12834. doi: 10.15252/emmm.202012834 (PMC7933818; doi:10.15252/emmm.202012834)
Supplement: Supplementary file 1 — Appendix [file EMMM-13-e12834-s001.pdf]

NF45/NF90-mediated rDNA transcription provides a novel target for immunosuppressant development

Hsiang-i Tsai[1,*], Xiaobin Zeng[2,*], Longshan Liu[3,*], Shengchang Xin[4], Yingyi Wu[1], Zhanxue Xu[1], Huanxi Zhang[3]， Gan Liu[1], Zirong Bi[3], Dandan Su[1], Min Yang[1], Changxi Wang[3], Jing Zhao[4], John E. Eriksson[5], Wenbin Deng[1#], Fang Cheng[1,#], Hongbo Chen[1,#]

Appendix

Appendix Figure S1

Appendix Figure S2

Appendix Figure S3

Appendix Figure S4

Appendix Figure S5

Appendix Figure S6

Appendix Figure S7

Appendix Figure S8

Appendix Table S1

Appendix Table S2

Appendix Table S3

Appendix Fig S1

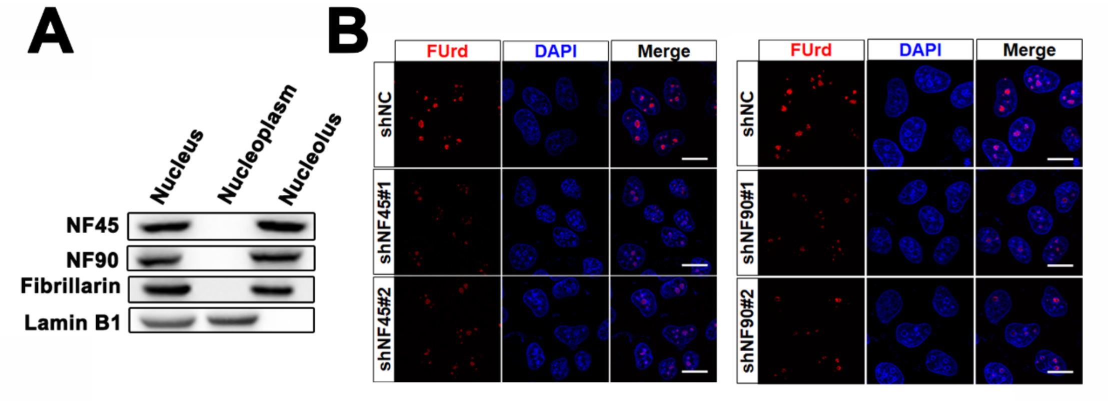

Appendix Figure S1. NF45/NF90 is a nucleolar protein complex and positively regulates rDNA transcription (related to Figure 1)**.**

A. HeLa cells were fractionated into nuclear, nucleoplasm and nucleolar fractions, and protein extracts were analyzed using western blot. Fibrillarin is a nucleolar marker; Lamin B1 is a nucleoplasm marker.

B. HeLa cells were silenced with negative control shRNA (shNC), NF45 shRNAs (shNF45#1 or sh4NF5#2) or NF90 shRNAs (shNF90#1 or shNF90#2), and then pulsed with FUrd for another 15 min in order to stain with an anti-BrdU (red). Scale bar, 10 μm.

Appendix Fig S2

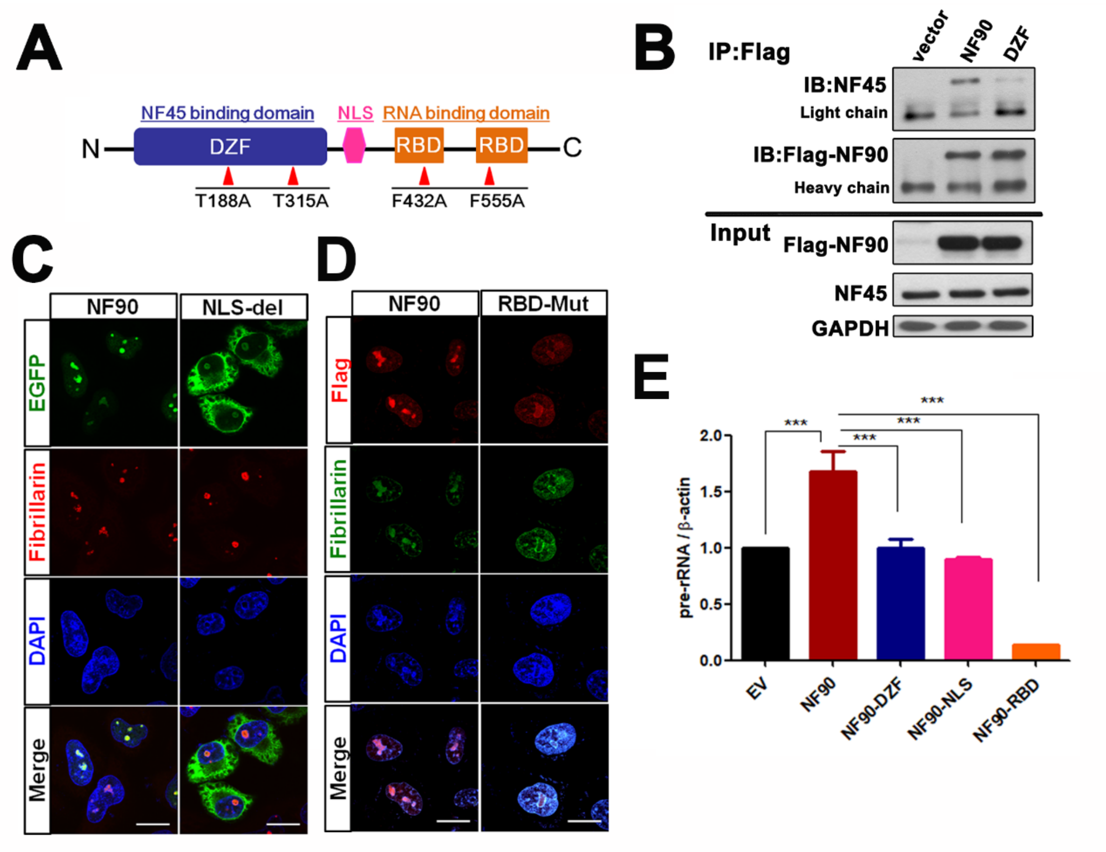

Appendix Figure S2. The rDNA transcriptional regulation of NF45/NF90 requires them to act as a complex and locate in the nucleolus. (related to Figure 1)**.**

A. Schematic diagram showing the domains of NF90 and its mutations (point mutations in DZF and RBD domains or NLS deleted mutation).

B. Co-IP analysis of NF45 with wild-type NF90 or DZF-mutated NF90.

C. Confocal images of HEK293 cells transfected with EGFP-NF90 or EGFP-NF90-NLS plasmid (fibrillarin is a nucleolar marker).

D. Confocal images of HEK293 cells transfected with FLAG-NF90 or FLAG-NF90-RBD plasmid. Scale bar, 10 μm.

E. HEK293 cells were transfected with an FLAG-NF90 vector or mutated vectors (DZF, NLS, or RBD) for 24 h, and then the total RNA was extracted from cells for qPCR analysis of pre-rRNA (n = 3) .

In all panels, Bar = mean ± SD. Statistical analysis by One-way ANOVA analysis followed by Tukey's test (E). (*** $p < 0.001$). Exact p values are reported in Appendix Table S2.

Appendix Fig S3

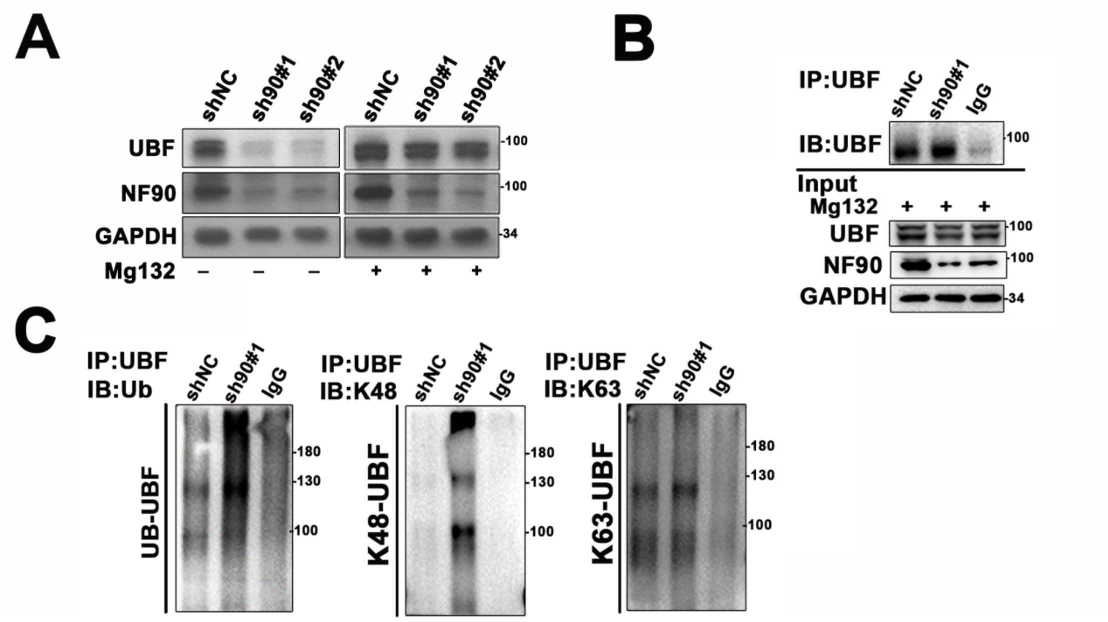

Appendix Figure S3. NF90 knockdown increased UBF ubiquitination (related to Figure 3)**.**

A. Western blot showing UBF and NF90 protein level in HEK293 cells infected with lentivirus containing NF90-specfic shRNAs (shNF90#1 and shNF90#2).

B. IP analysis of UBF in HEK293 infected with lentivirus containing shNF90#1.

C. IP analysis of UBF ubiquitination in HEK293 infected with lentivirus containing shNF90#1.

Appendix Fig S4

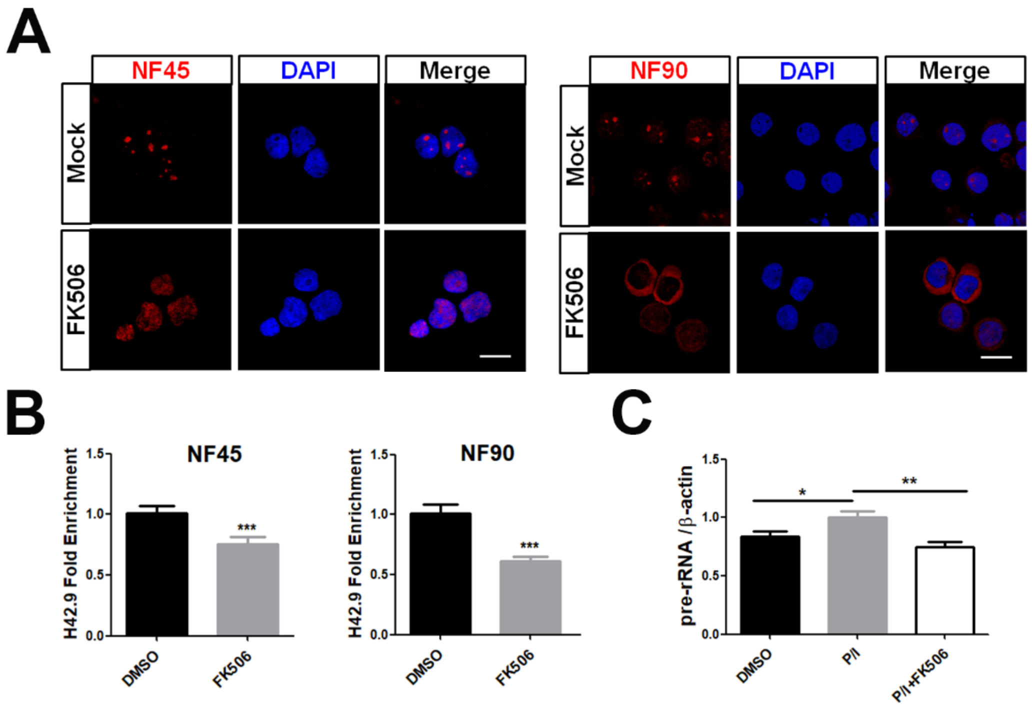

Appendix Figure S4. FK506 affects the subcellular localization of NF45/NF90 and rDNA transcription in T cells (related to Figure 4)**.**

A. Confocal images show the localization of NF45 (left panel) or NF90 (right panel) in P/I-stimulated $CD3^+$ T cells treated with or without FK506. Scale bar, 10 μm.

B. The binding of NF45 and NF90 to rDNA in P/I-stimulated $CD3^+$ T cells treated with or without FK506 (n = 3).

C. qPCR analysis of pre-rRNA in $CD3^+$ cells treated with P/I and a combination of P/I and FK506 (n = 3).

In all panels, Bar = mean ± SD. Statistical analysis by unpaired Student T-test (B). One-way ANOVA analysis followed by Tukey's test (C). (** $p < 0.01$, *** $p < 0.001$). Exact p values are reported in Appendix Table S2.

Appendix Fig S5

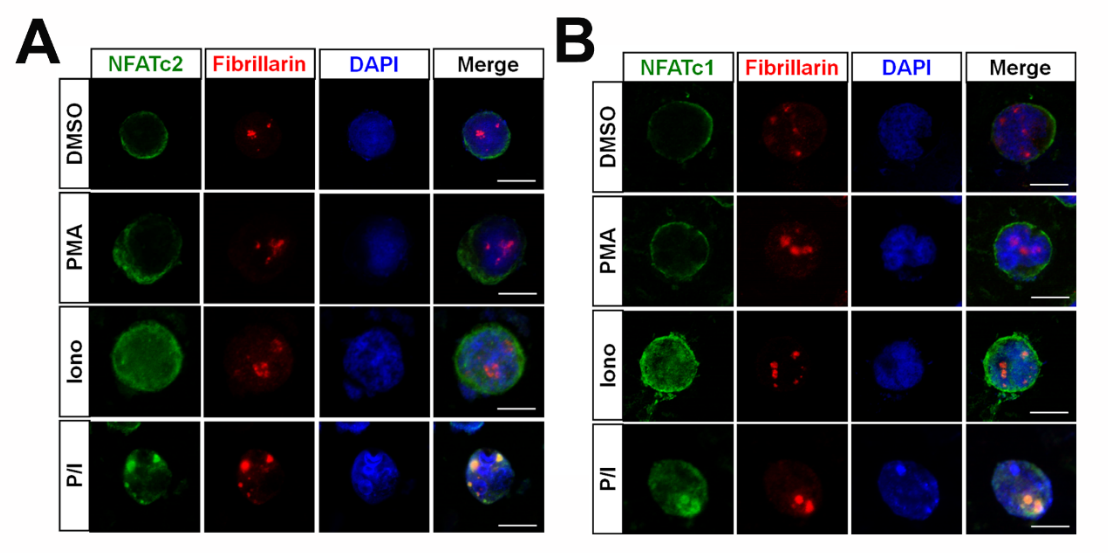

Appendix Figure S5. NFATc2 and NFATc1 translocated to the nucleolus upon Iono or P/I stimulation (related to Figure 4)**.**

A-B. Confocal images to show the subcellular localization of NFATc2 (A) or NFATc1 (B) in Jurkat cells treated with DMSO, PMA, Ionomycin or P/I. Scale bar, 10 μm.

Appendix Fig S6

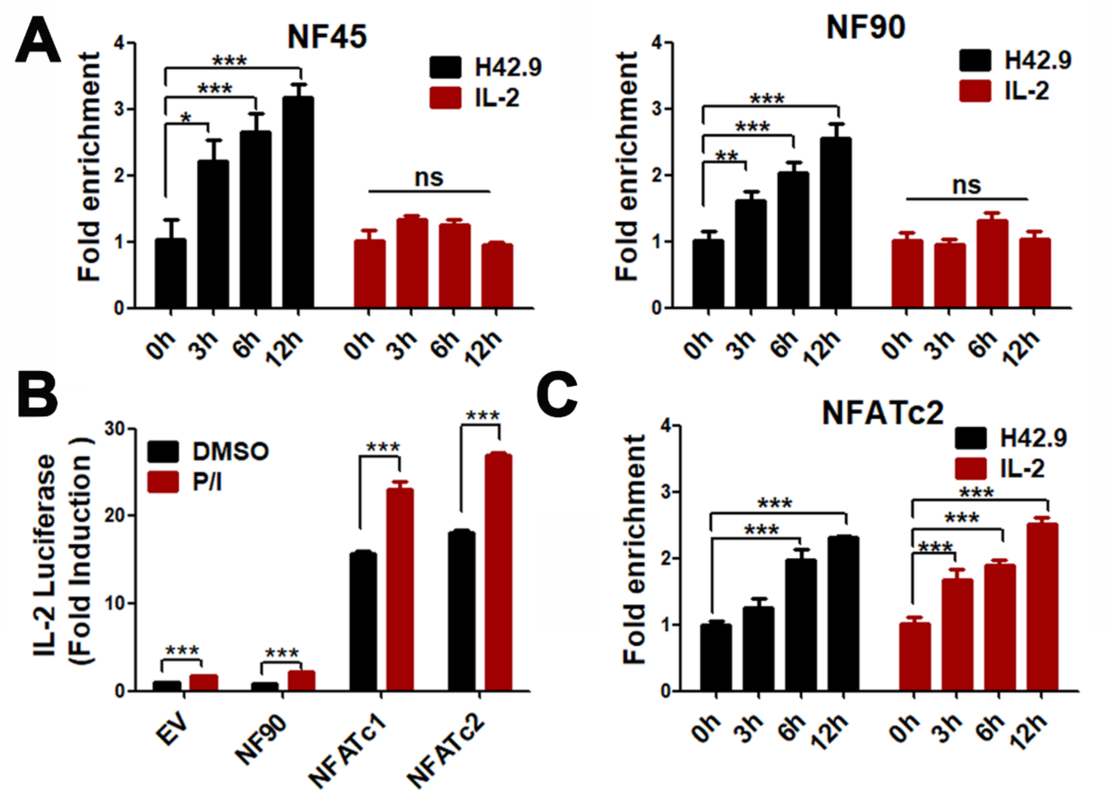

Appendix Figure S6. The regulation functions of NF45/NF90 and NFATc1 or NFATc2 on rRNA transcription and IL-2 expression.

A. The binding ability of NF45 or NF90 to the rDNA or IL-2 loci detected by a ChIP assay in $CD3^{+}$ cells stimulated with or without of P/I for the indicated times (n = 3).

B. Jurkat cells were transfected with empty vector (EV), FLAG-NF90, V5-NFATc2 or V5-NFATc1 in combination with IL-2-Luc reporter plasmids for 24 h, and then stimulated with or without of P/I for 12 h (n = 3) , The relative luciferase activity of IL-2 was detected.

C. The binding ability of NFATc2 to the rDNA or IL-2 loci detected by a ChIP assay in $CD3^{+}$ cells stimulated with or without of P/I for the indicated times (n = 3).

In all panels, Bar = mean ± SD. Statistical analysis by unpaired Student T-test (C). One-way ANOVA analysis followed by Tukey's test (A, B). (** $p < 0.01$, *** $p < 0.001$). Exact p values are reported in Appendix Table S2.

Appendix Fig S7

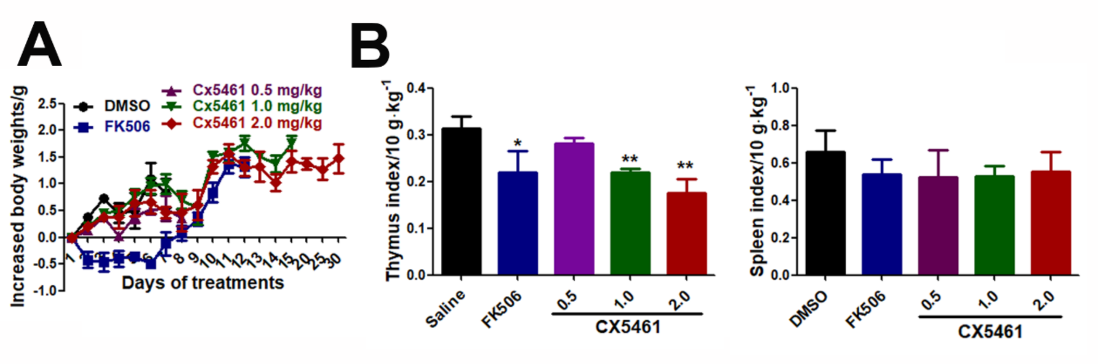

Appendix Figure S7. The effects of CX5461 on the body weight, spleen weight and thymus weight of skin-graft mice (related to Figure 6)**.**

A. Plots of the body weight of skin-graft mice after injecting with DMSO, FK506 or different doses of CX5461.

B. The thymuses and spleens of each group were weighed at the end point of the study (n = 5). In all panels, Bar = mean ± SD. Statistical analysis by One-way ANOVA analysis followed by Tukey's test (B). (* $p < 0.05$, *** $p < 0.001$). Exact p values are reported in Appendix Table S2.

Appendix Fig S8

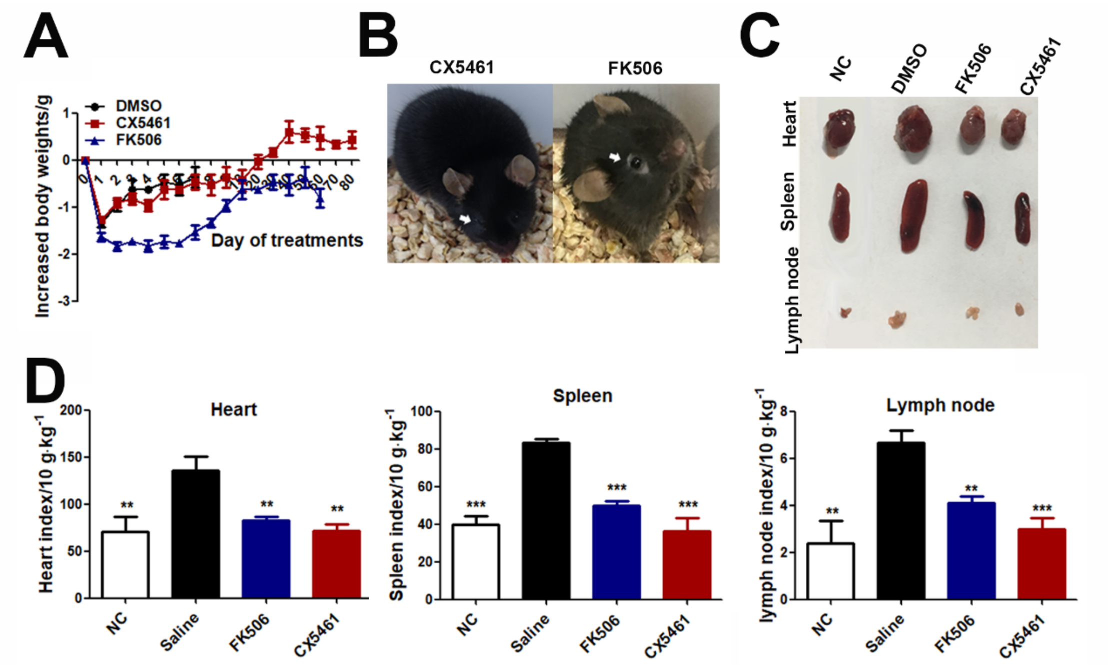

Appendix Figure S8. The effects of CX5461 on the body weight, hair loss, and spleen and thymus weights of heart-transplant mice (related to Figure 6)**.**

A. Plots of body weights of heart-transplant mice after injecting with DMSO, FK506 or CX5461.

B. Images highlighting the hair loss of heart-transplant mice after injecting with FK506 or CX5461 at Day 7.

C-D. Images (C) and weights (D) of the viscera of heart-grafts, spleens and lymph nodes of each group at the end point of the study (n = 5).

In all panels, Bar = mean ± SD. Statistical analysis by One-way ANOVA analysis followed by Tukey's test (D). (** $p < 0.01$, *** $p < 0.001$). Exact p values are reported in Appendix Table S2.

Appendix Table S1. The demographic and baseline clinical characteristics of the study in kidney transplant patients

**Variables**	**Total (n = 23)**	**ABMR (n = 8)**	**TCMR (n = 8)**	**stable (n = 7)**	***P***
Donors					
Types, n (%)					**0.005**
DD	13 (57)	2 (25)	8 (100)	3 (43)	
LD	10 (43)	6 (75)	0 (0)	4 (57)	
Recipients					
Gender, n (%)					**0.017**
Female	9 (39)	1 (12)	2 (25)	6 (86)	
Male	14 (61)	7 (88)	6 (75)	1 (14)	
Age (year), Median (IQR)	33.81 (31.40, 39.65)	37.84 (34.13, 48.32)	32.97 (30.31, 41.70)	31.72 (30.76, 33.50)	0.102
Weight (kg), Median (IQR)	52.0 (43.8, 63.8)	63.3 (52.0, 64.0)	54.3 (44.9, 63.3)	42.5 (41.4, 50.0)	0.138
Previous transplantation, n (%)					0.494
0	20 (87)	8 (100)	6 (75)	6 (86)	
1	3 (13)	0 (0)	2 (25)	1 (14)	
Days from transplant to sample, Median (IQR)	366 (140, 582)	1474 (633, 2353)	128 (103, 149)	376 (369, 378)	**0.001**
CNIs *, n (%)					1.000
CsA	1 (4)	0 (0)	1 (12)	0 (0)	
FK506	22 (96)	8 (100)	7 (88)	7 (100)	
FK506 C_0 (ng/ml), Median (IQR)	6.6 (5.3, 7.8)	7.7 (7.2, 8.5)	5.7 (5.1, 6.6)	6.3 (5.6, 8.3)	0.161
CsA C_0 (ug/L) #	—	—	127.5	—	—
White blood cell count (10^9/L), Median (IQR)	6.99 (5.98, 9.21)	7.00 (6.22, 8.97)	7.44 (6.39, 9.86)	6.41 (5.98, 8.25)	0.694
Lymphocyte count (10^9/L), Mean ± SD	1.41 ± 0.68	1.13 ± 0.37	1.53 ± 0.82	1.61 ± 0.75	0.339
Neutrophil (10^9/L), Median (IQR)	4.65 (3.74, 6.85)	5.05 (4.30, 7.62)	6.06 (4.29, 6.87)	3.80 (3.54, 5.65)	0.408
Monocyte count (10^9/L), Mean ± SD	0.61 ± 0.21	0.62 ± 0.18	0.68 ± 0.29	0.52 ± 0.14	0.363

CNIs, Calcineurin inhibitors; FK506, Tacrolimus; CsA, Cyclosporin A; LD, Living donor; DD, Death donor. * Standard maintenance immunosuppression, including CNI, prednisolone and mycophenolic acid was applied to all patients. # Only one patient received cyclosporine A, and no standard deviation of CsA C_0 was provided.

Appendix Table S3. List of qPCR primers

Gene	Forward primer sequence 5'→3'	Reverse primer sequence 5'→3'
Human *Actb*	**GAACGGTGGTGTGTCGTTC**	**GCGTCTCGTCTCGTCTCACT**
Human pre-rRNA	**GAACGGTGGTGTGTCGTTC**	**GCGTCTCGTCTCGTCACT**
Mouse *Actb*	**GGCTGTATTCCCCTCCATCG**	**CCAGTTGGTAACAATGCCATGT**
Mouse pre-rRNA	**CTCCTGTCTGTGGTGTCCAA**	**GCTGGCAGAACGAGAAGAAC**
Human H0	**GGTATATCTTTCGCTCCGAG**	**GACGACAGGTCGCCAGAGGA**
Human H13	**ACCTGGCGCTAAACCATTCGT**	**GGACAAACCCTTGTGTCGAGG**
Human H23	**CCTTCCACGAGAGTGAGAAGC**	**TCGACCTCCCGAAATCGTACA**
Human H42.9	**CCCGGGGGAGGTATATCTTT**	**CCAACCTCTCCGACGACA**
Human IL-2 promoter	**TAAGTGTGGGCTAACCCGA**	**AAGGAGCACAAGTGTCAATGTGA**

Appendix Table S3. List of exact *p*-values

Figure	Panel	Sub-pancel	Compared groups	*P*-value
Figure 1	B	shNF45	shNF45#1 vs NC<br>shNF45#2 vs NC	0.0202<br>0.0125
		shNF90	shNF90#1 vs NC<br>shNF90#2 vs NC	<0.001<br><0.001
	C	NF45	NF45 vs EV	<0.001
		NF90	NF90 vs EV	<0.001
	E	shNF45	shNF45#1 vs NC<br>shNF45#2 vs NC	<0.001<br><0.001
		shNF90	shNF90#1 vs NC<br>shNF90#2 vs NC	<0.001<br><0.001
	F	NF45	NF45 vs EV	<0.001
		NF90	NF90 vs EV	<0.001
Figure 2	E	NF90-	Mutant vs WT	<0.001
		NF90+	Mutant vs WT	<0.001
		WT	NF90+ vs NF90-	<0.001
Figure 3	E	NF45	3 h vs 0 h<br>6 h vs 0 h	0.0060<br><0.001
		NF90	3 h vs 0 h<br>6 h vs 0 h	0.0053<br><0.001
	F	NF45	NF45 vs EV	<0.001
		NF90	NF90 vs EV	<0.001
Figure 4	A	NF45	P/I vs DMSO	0.0283
		NF90	P/I vs DMSO	<0.001
	B		P/I vs DMSO	0.0177
	C	shNC	P/I vs DMSO	<0.001
	D	shNC	P/I vs DMSO	<0.001
	G	DMSO	NFATc2 vs IgG	<0.001
		P/I	NFATc2 vs IgG	<0.001
	H	WT	EV vs NF90	<0.001
		PI	NF90 vs EV<br>NFATc2 vs EV<br>NF90+NFATc2 vs NF90<br>NF90+NFATc2 vs NFAT	<0.001<br><0.001<br><0.001<br><0.001
	I	shNC	P/I vs DMSO	<0.001
Figure 5	B		shNF45#1 vs NC<br>shNF45#2 vs NC<br>shNF90#1 vs NC<br>shNF90#2 vs NC	<0.001<br><0.001<br><0.001<br><0.001
	C		shNF45#1 vs NC<br>shNF45#2 vs NC	0.0278<br>0.0147

			shNF90#1 vs NC<br>shNF90#2 vs NC	0.0027<br>0.0113
	G		DMSO vs P/I<br>P/I+FK506 vs P/I<br>P/I+CX5461 vs P/I	<0.001<br><0.001<br><0.001
	H		DMSO vs P/I<br>P/I+FK506 vs P/I<br>P/I+CX5461 vs P/I	<0.001<br>0.0366<br><0.001
Figure 6	A	Skin allograft<br>Heart allograft	Rejection vs NC<br>Rejection vs NC	<0.001<br><0.001
	B		TCMR vs Stable	0.0042
	G		FK506 vs DMSO<br>CX54610 1.0 vs DMSO<br>CX54610 2.0 vs DMSO	0.0267<br>0.0113<br>0.0061
	H		FK506 vs DMSO<br>CX54610 0.5 vs DMSO<br>CX54610 1.0 vs DMSO<br>CX54610 2.0 vs DMSO	0.0069<br>0.0197<br>0.0035<br>0.0036
	I		FK506 vs DMSO<br>CX5461 vs DMSO	0.0099<br><0.001
	L		NC vs DMSO<br>FK506 vs DMSO<br>CX5461 vs DMSO	<0.001<br><0.001<br><0.001
	M		NC vs DMSO<br>FK506 vs DMSO<br>CX5461 vs DMSO	<0.001<br>0.0070<br><0.001
	N		NC vs DMSO<br>FK506 vs DMSO<br>CX5461 vs DMSO	<0.001<br><0.001<br><0.001
Appendix Figure S2	E		NF90 vs EV<br>NF90-DZF vs EV<br>NF90-NLS vs EV<br>NF90-RBD vs EV	<0.001<br><0.001<br><0.001<br><0.001
Appendix Figure S4	B	NF45	FK506 vs DMSO	<0.001
		NF90	FK506 vs DMSO	<0.001
	C		DMSO vs P/I<br>P/I+FK506 vs P/I	0.0177<br>0.0034
Appendix Figure S6	A	NF45- H42.9	3 h vs 0 h	0.0140
			6 h vs 0 h	<0.001
			12 h vs 0 h	<0.001
		NF90- H42.9	3 h vs 0 h	0.0056
			6 h vs 0 h	<0.001
			12 h vs 0 h	<0.001

	B	NFATc2- H42.9	6 h vs 0 h	<0.001
			12 h vs 0 h	<0.001
		NFATc2- IL-2	3 h vs 0 h	<0.001
			6 h vs 0 h	<0.001
			12h vs 0h	<0.001
	C	EV	PI vs DMSO	<0.001
		NF90	PI vs DMSO	<0.001
		NFATc1	PI vs DMSO	<0.001
		NFATc2	PI vs DMSO	<0.001
Appendix Figure S7	B	Thymus index	FK506 vs DMSO<br>CX5461 1.0 vs DMSO<br>CX5461 2.0 vs DMSO	0.0378<br>0.0038<br>0.0039
Appendix Figure S8	D	Heart	NC vs DMSO<br>FK506 vs DMSO<br>CX5461 vs DMSO	0.0070<br>0.0049<br>0.0031
		Spleen	NC vs DMSO<br>FK506 vs DMSO<br>CX5461 vs DMSO	<0.001<br><0.001<br><0.001
		Lymph node	NC vs DMSO<br>FK506 vs DMSO<br>CX5461 vs DMSO	0.0026<br>0.0021<br><0.001
