# Supplementary material for: NF45/NF90‐mediated rDNA transcription provides a novel target for immunosuppressant development
Source: EMBO Mol Med. 2021 Feb 8;13(3):e12834. doi: 10.15252/emmm.202012834 (PMC7933818; doi:10.15252/emmm.202012834)

Appendix Figure S5A

DMSO

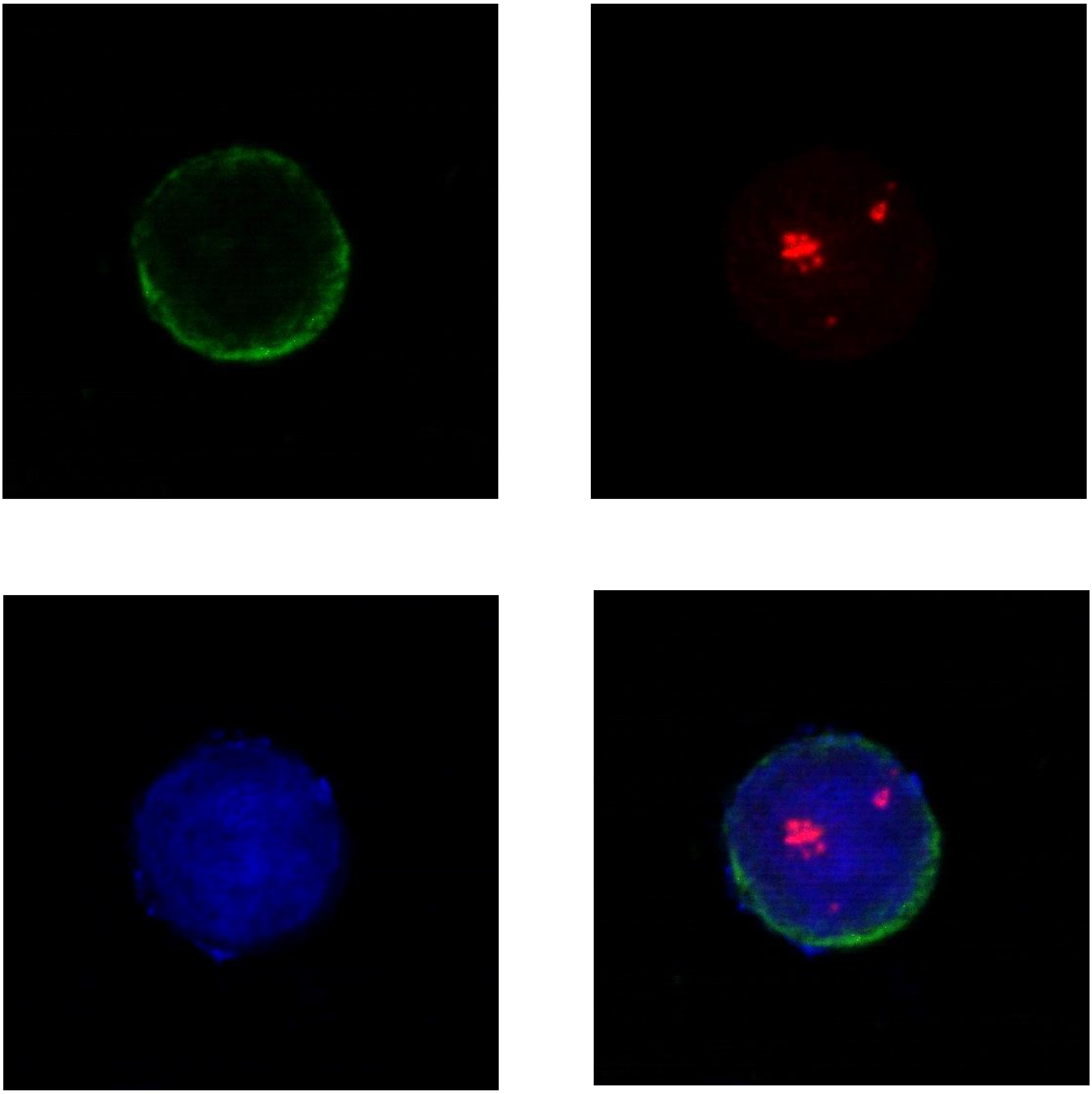

PMA

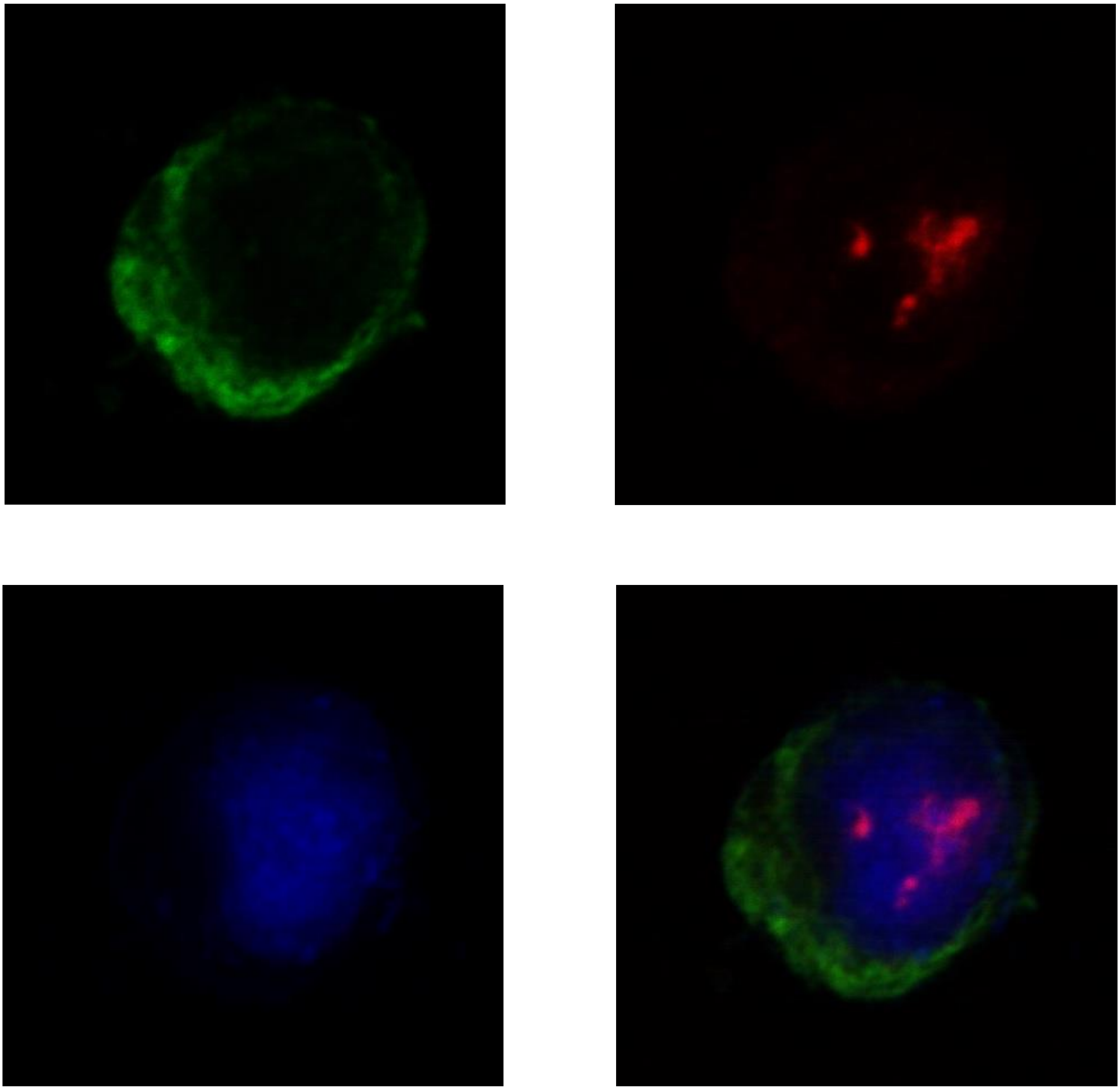

Ionomycin

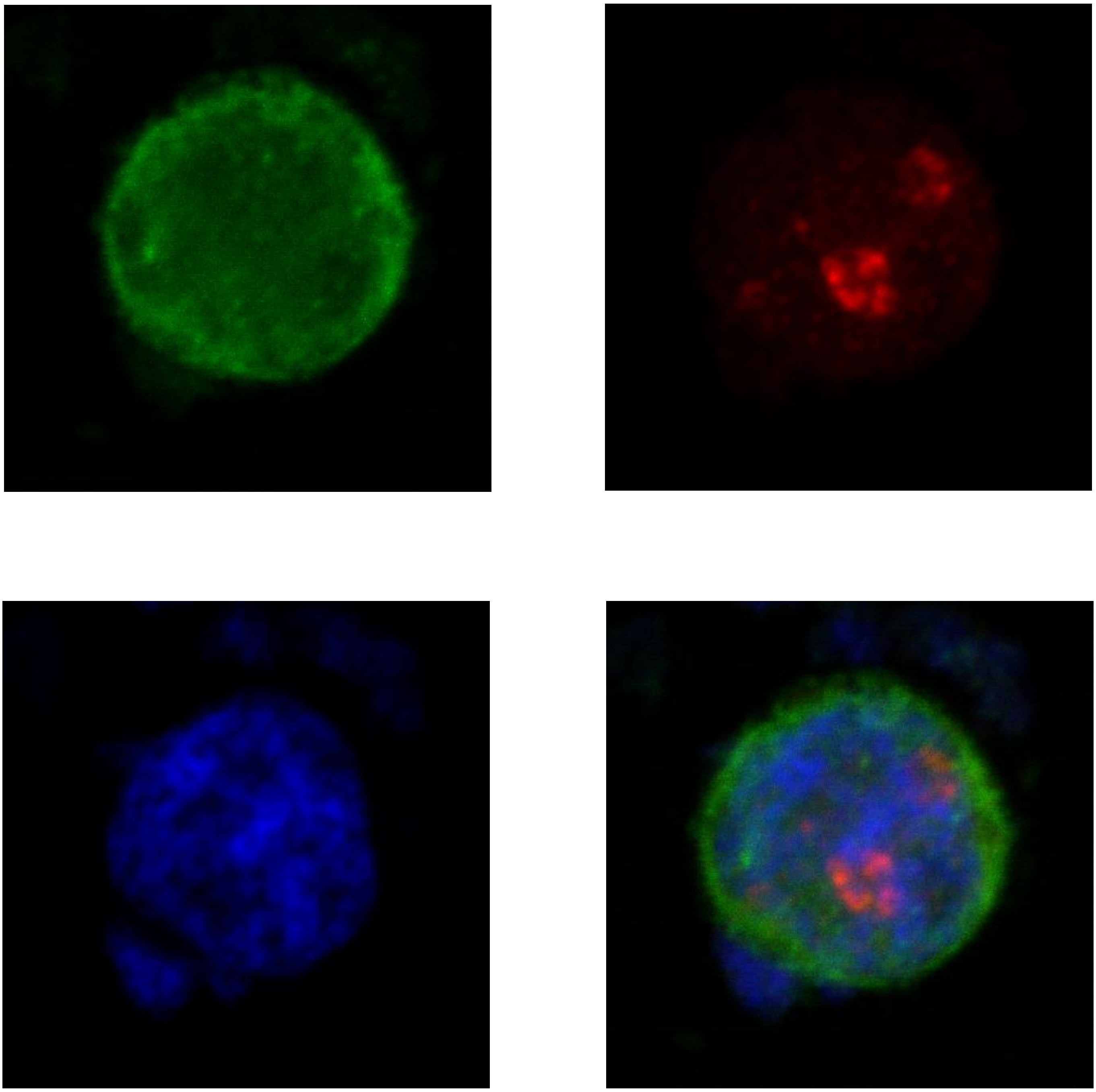

PMA+Ionomycin

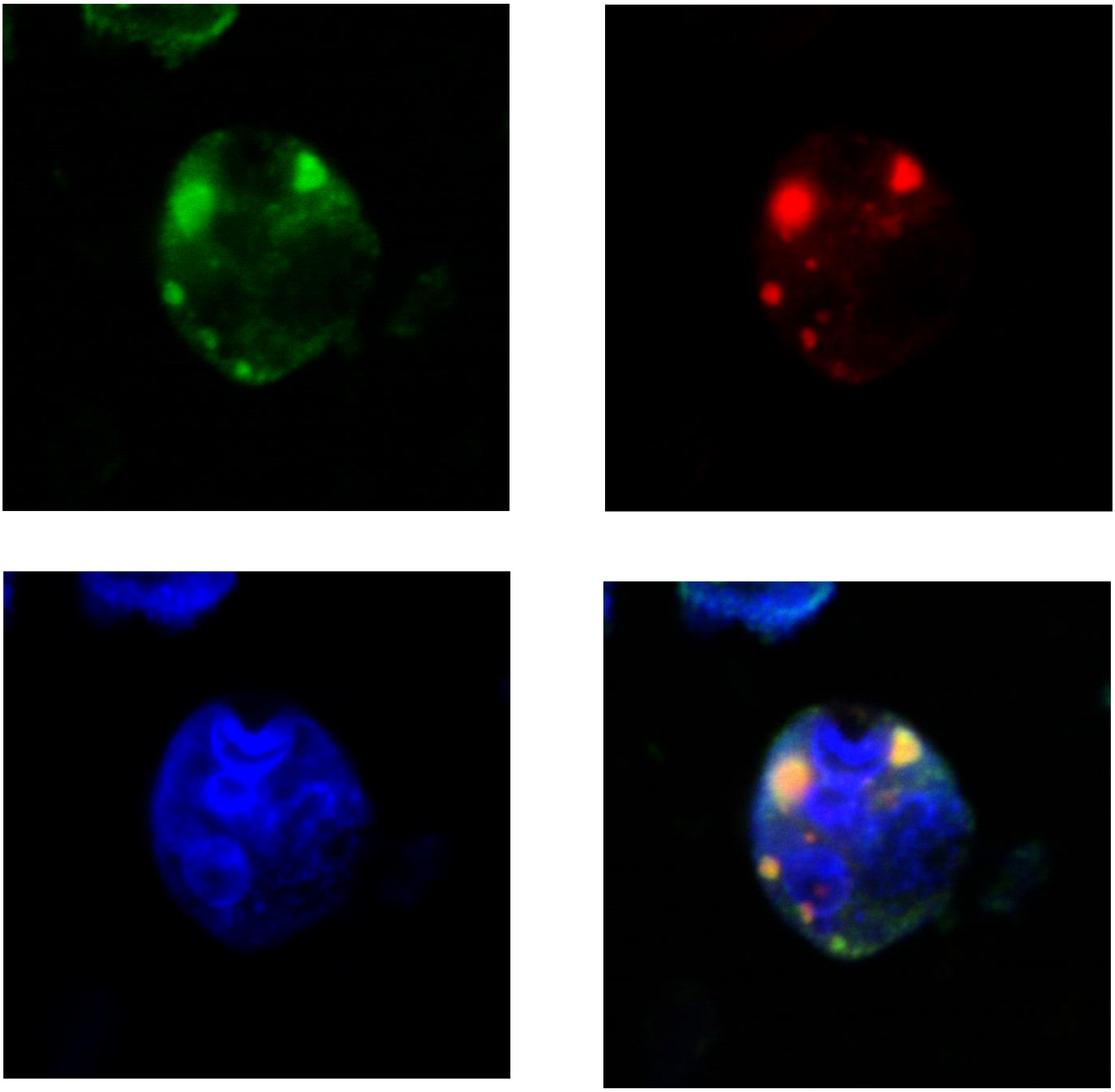

Appendix Figure S5B

DMSO

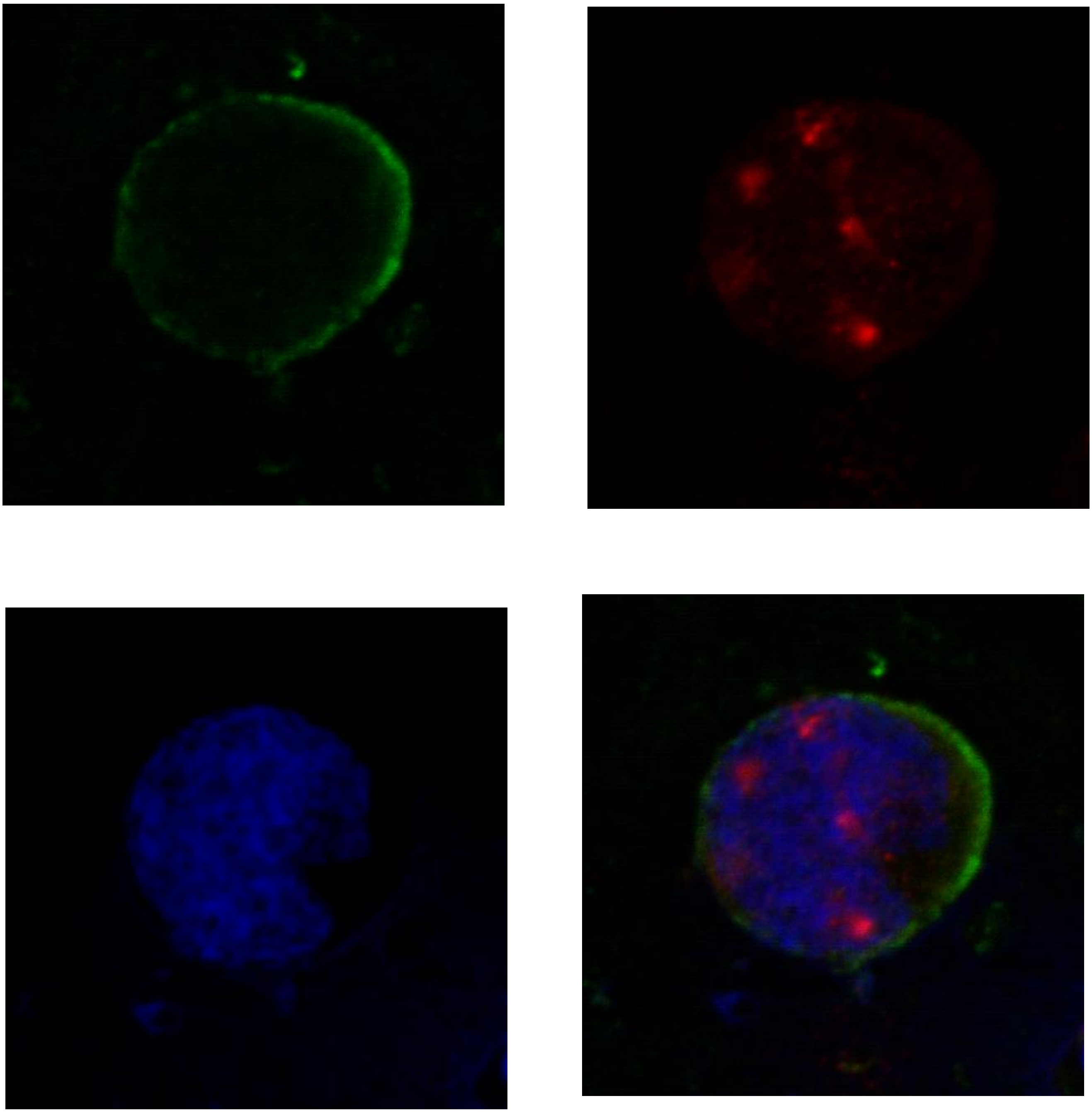

PMA

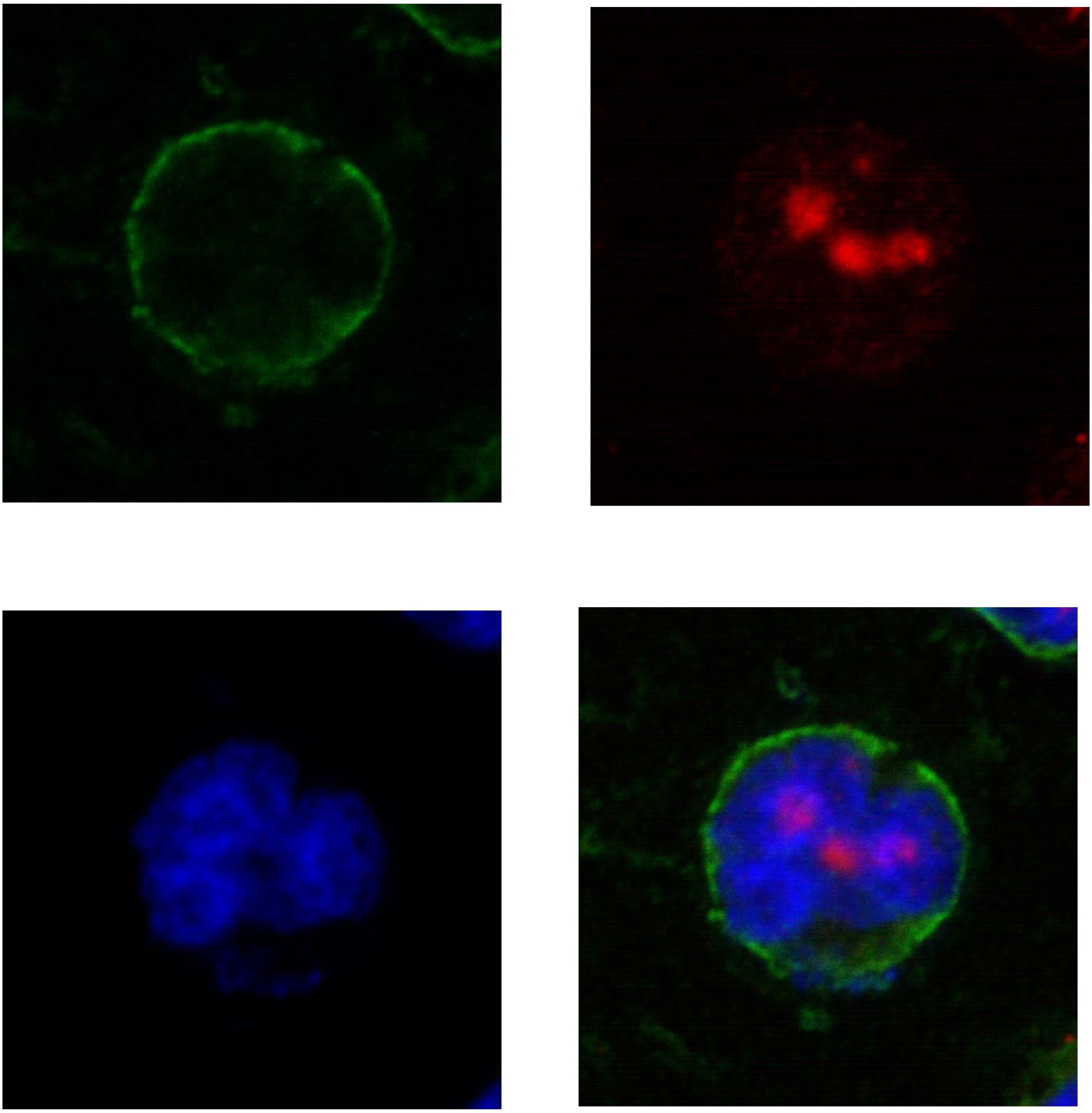

Ionomycin

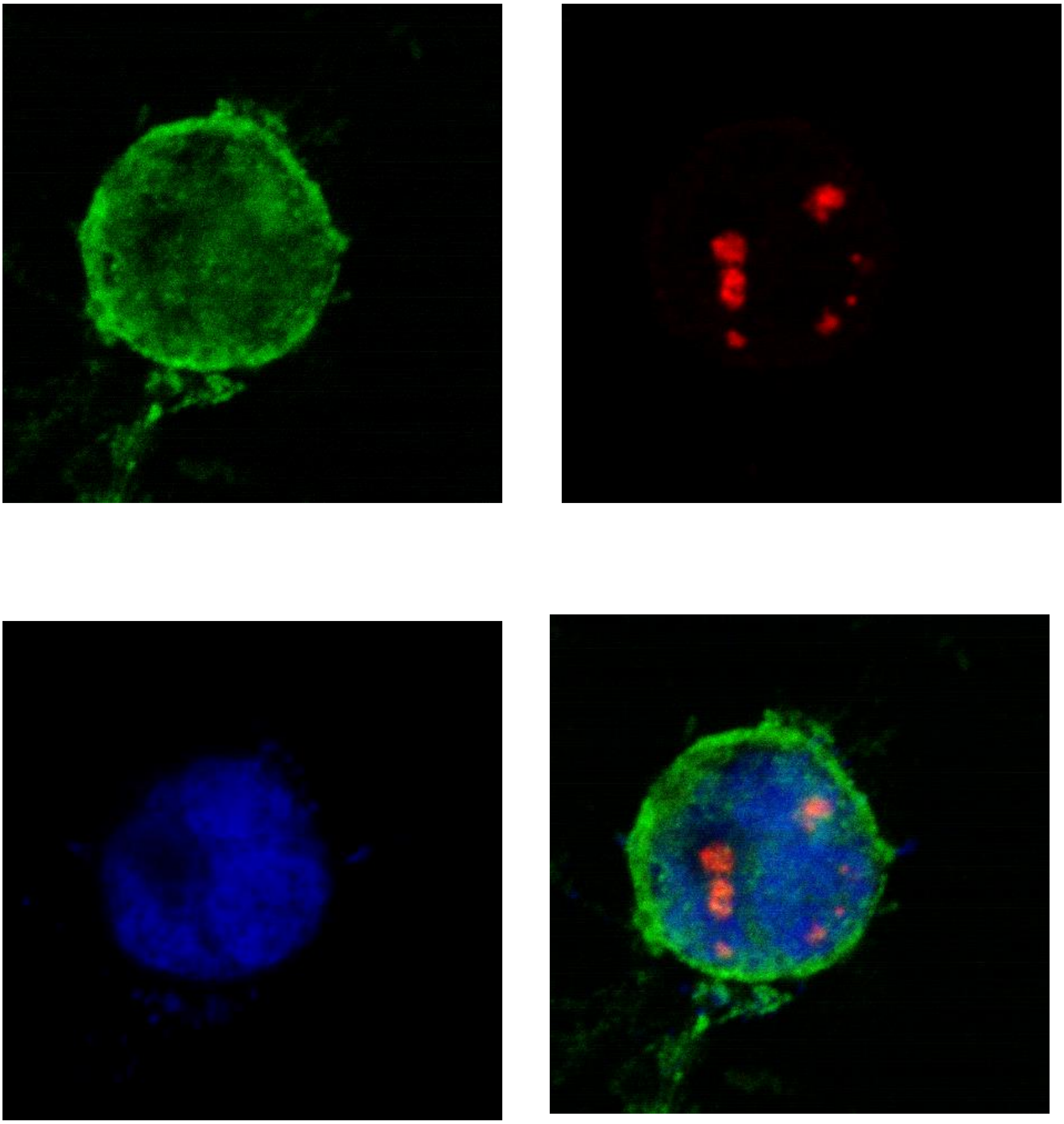

PMA+Ionomycin

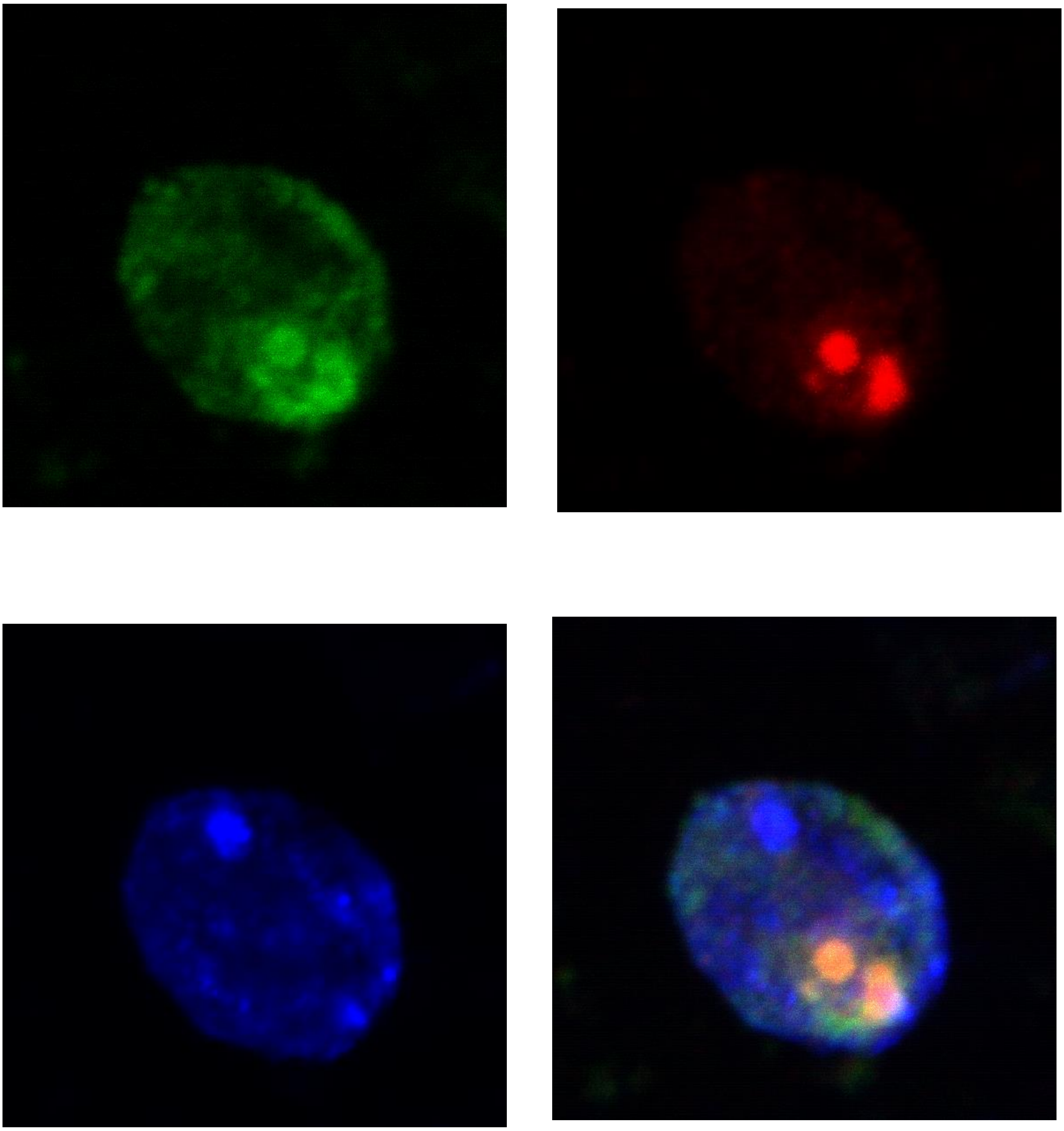

Supplement: Supplementary file 2 — Source Data for Appendix [file EMMM-13-e12834-s008.zip › Appendix_source_data/Source Data For Appendix Figure S5.pdf]
