# Supplementary figures and images for: NF45/NF90‐mediated rDNA transcription provides a novel target for immunosuppressant development
Source: EMBO Mol Med. 2021 Feb 8;13(3):e12834. doi: 10.15252/emmm.202012834 (PMC7933818; doi:10.15252/emmm.202012834)

Appendix Figure S2C

NF90

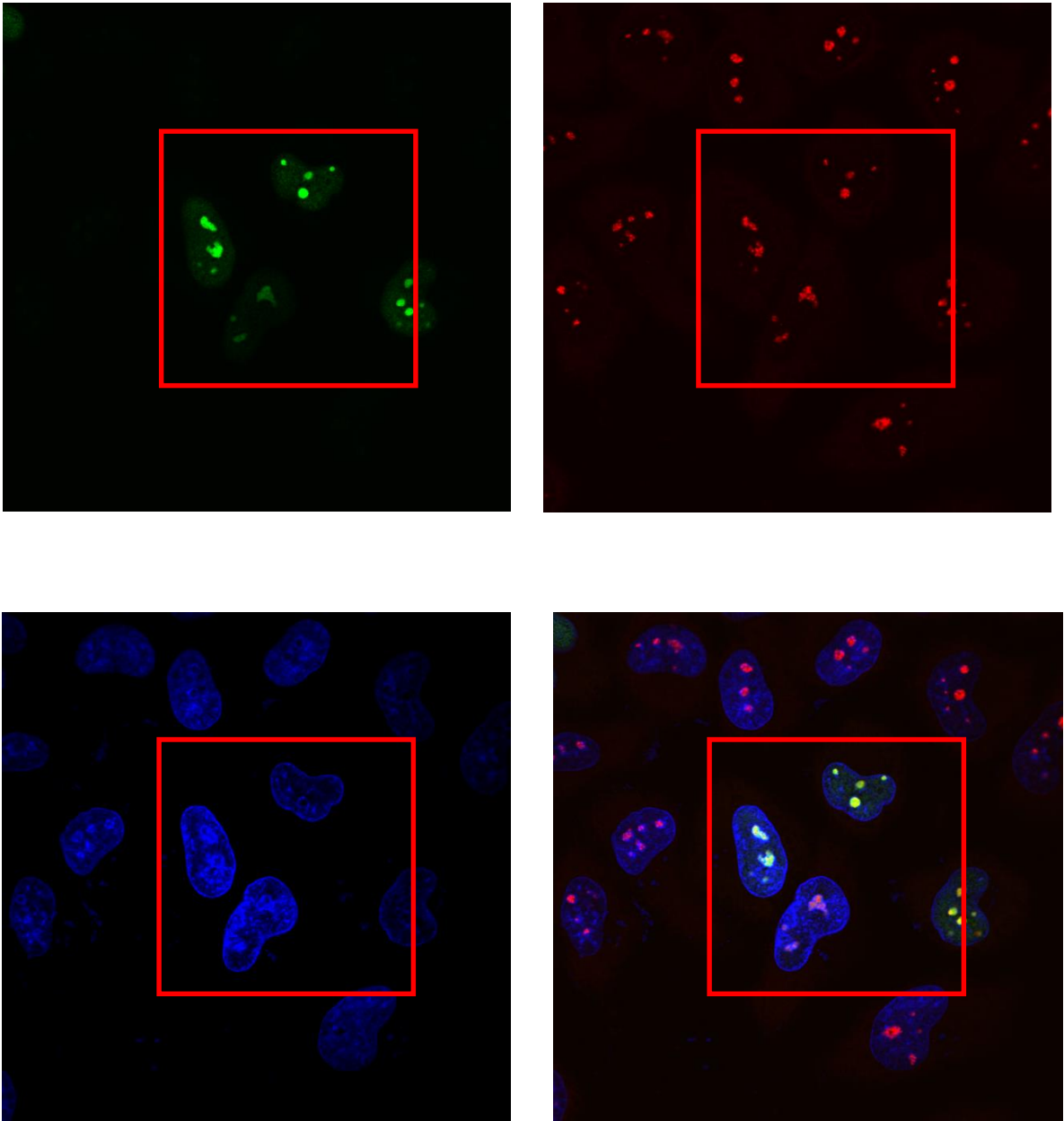

NLS-del

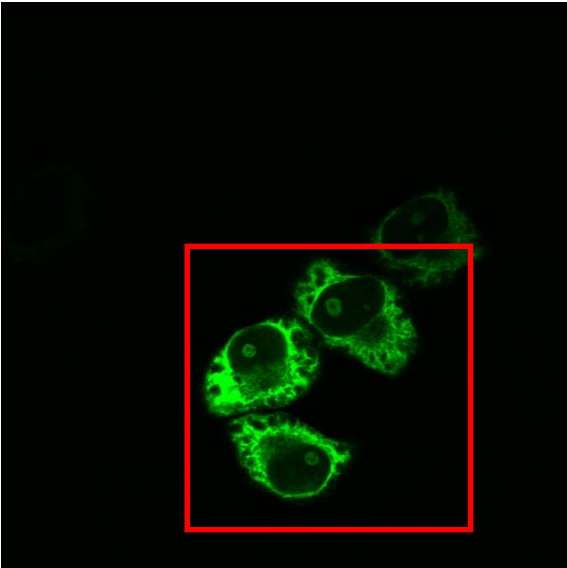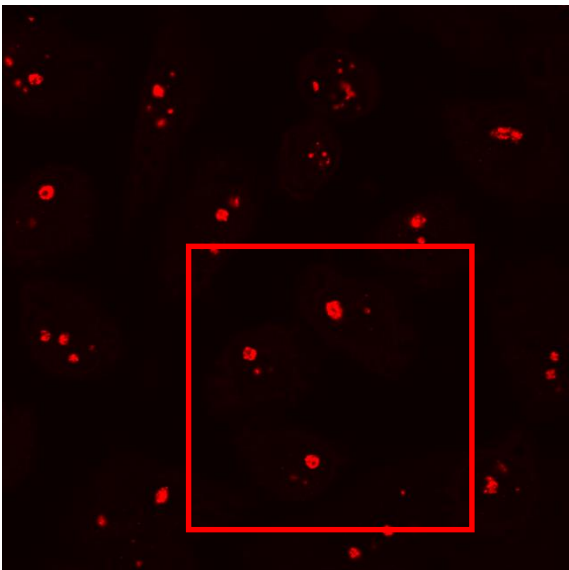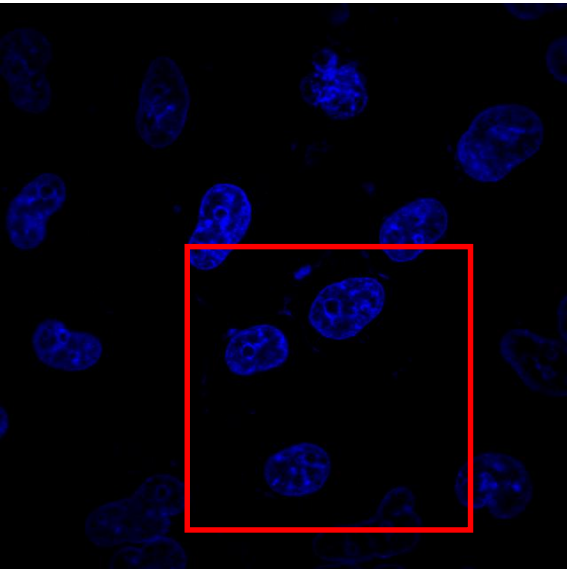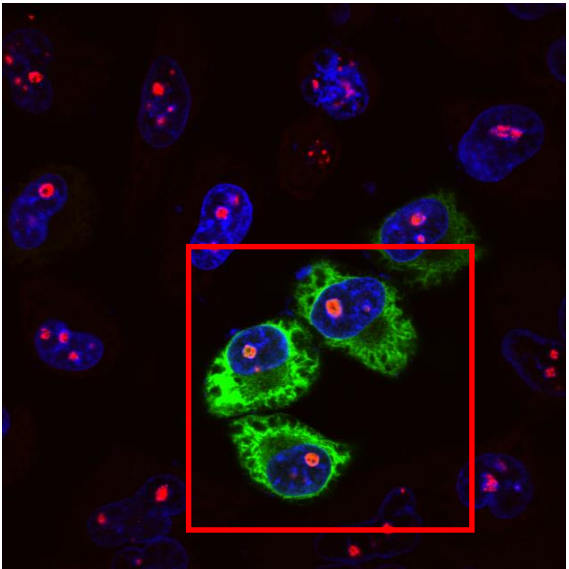

Appendix Figure S2D

NF90

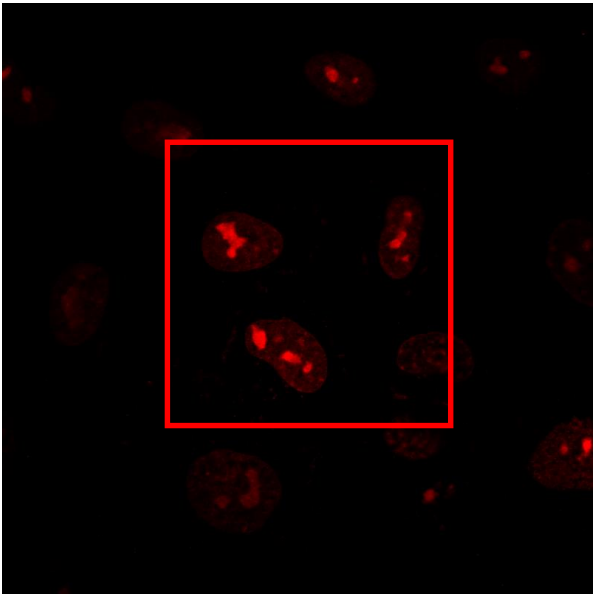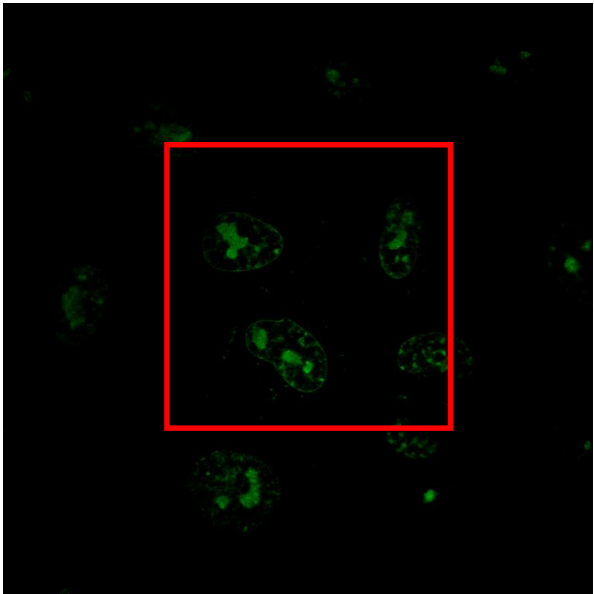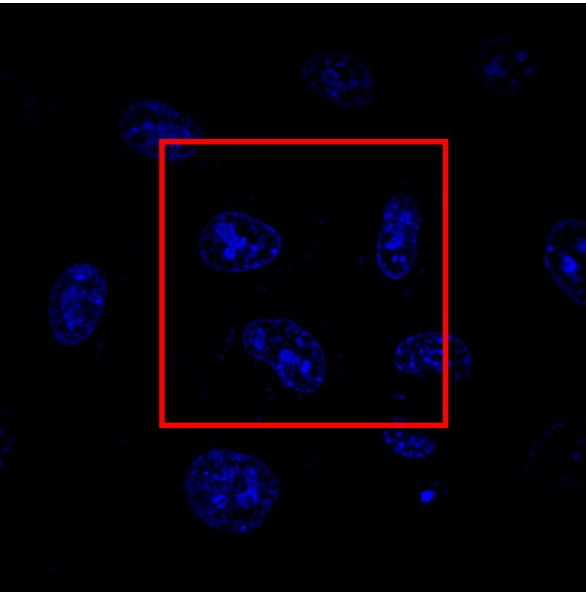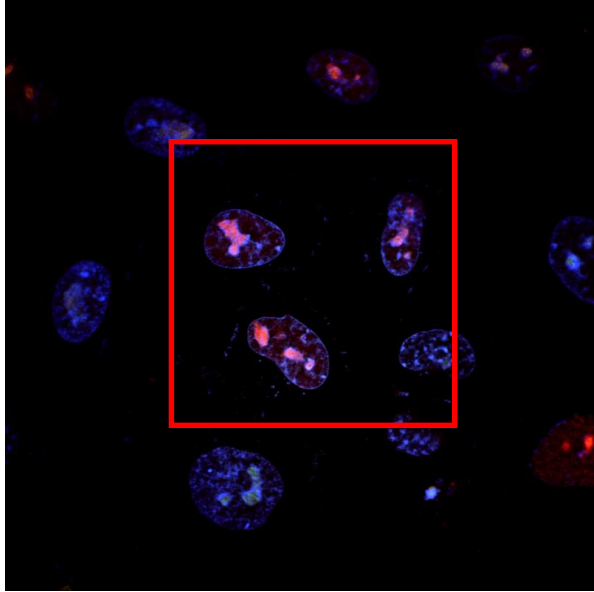

## RBD-Mut

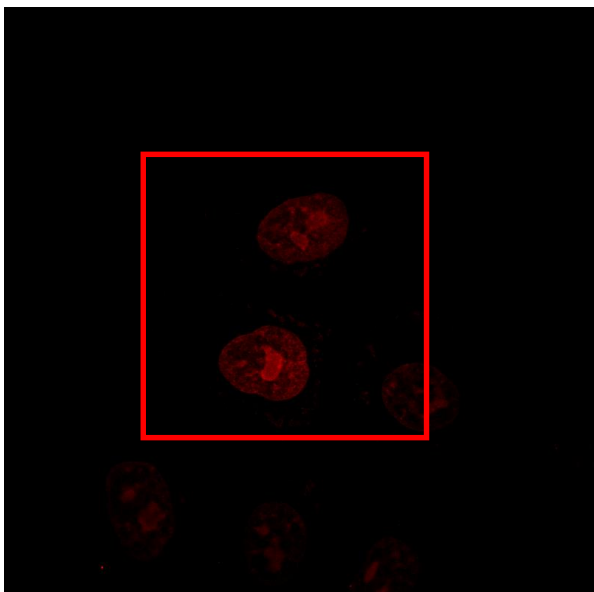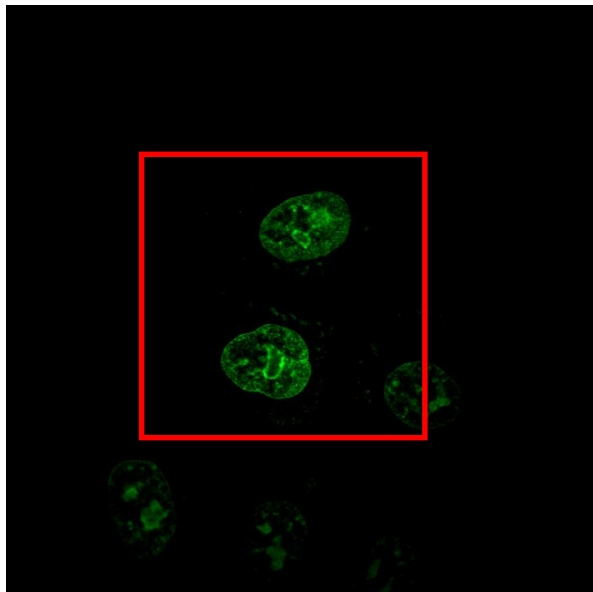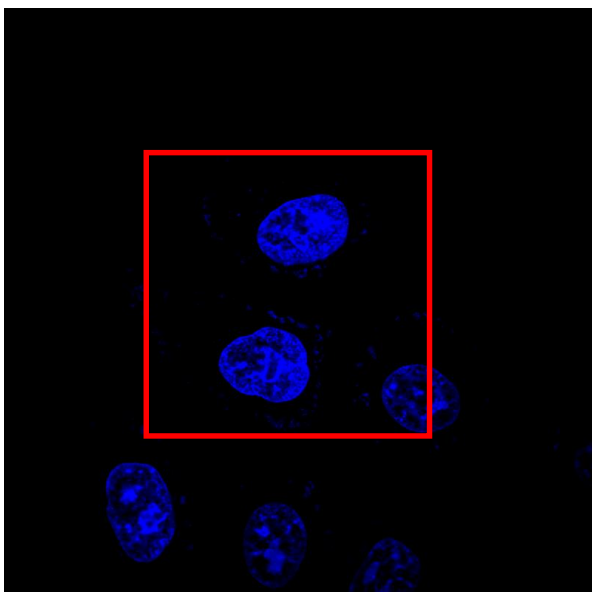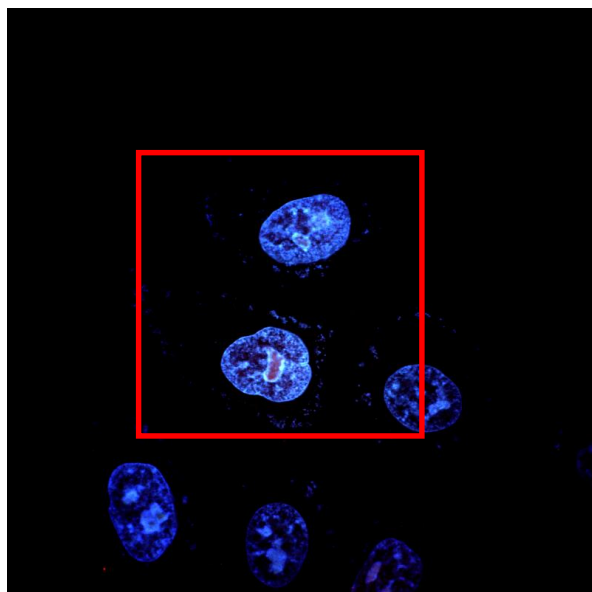

Supplement: Supplementary file 2 — Source Data for Appendix [file EMMM-13-e12834-s008.zip › Appendix_source_data/Source Data For Appendix Figure S2.pdf]

Appendix Figure S1B-left

shNC

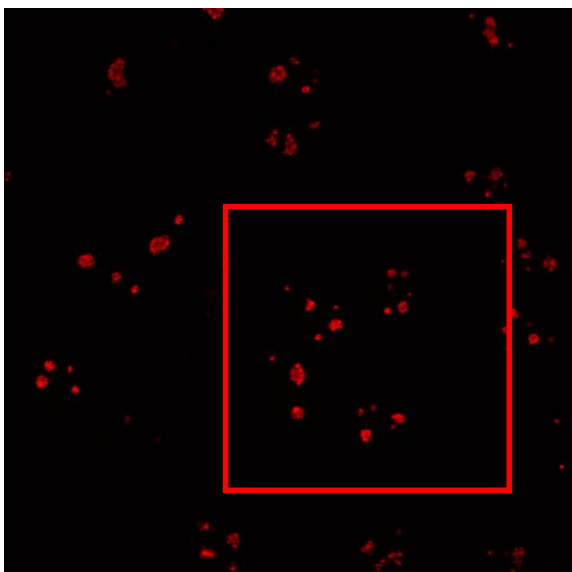

shNF45#1

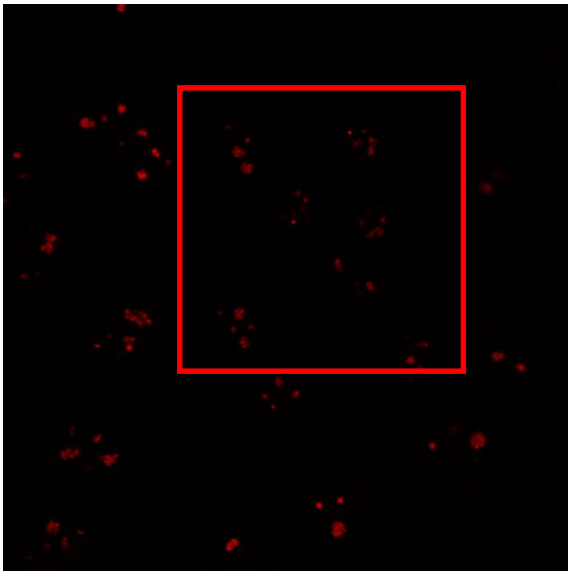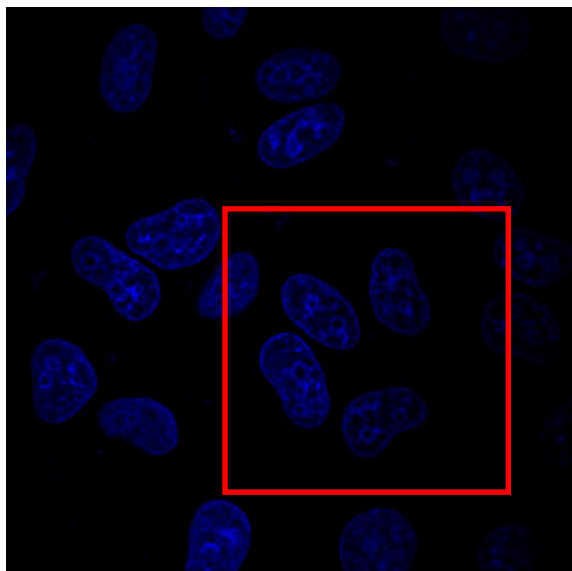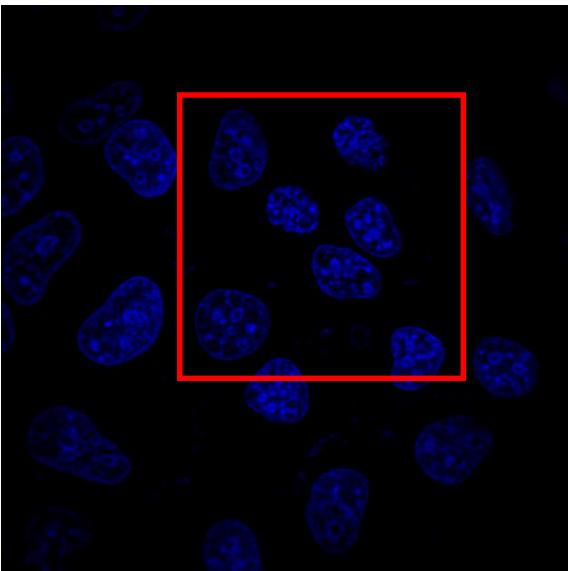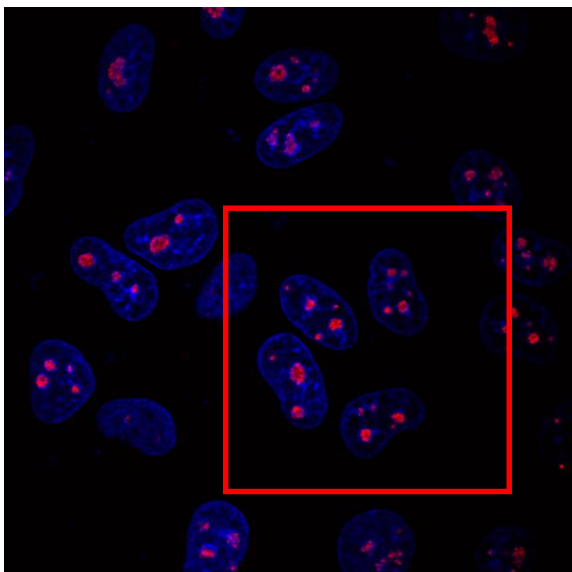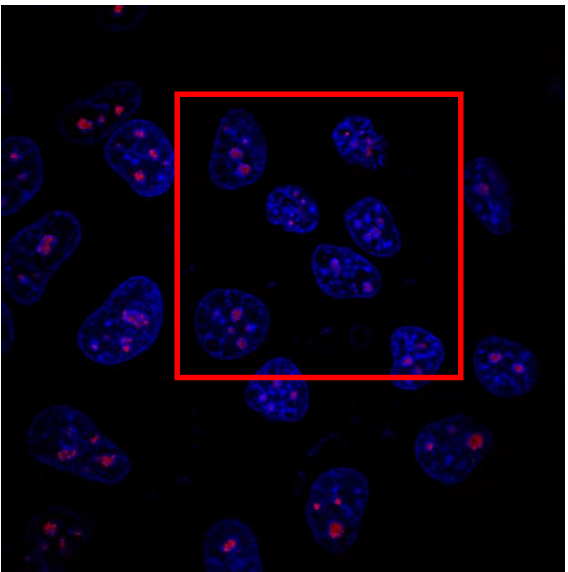

shNF45#2

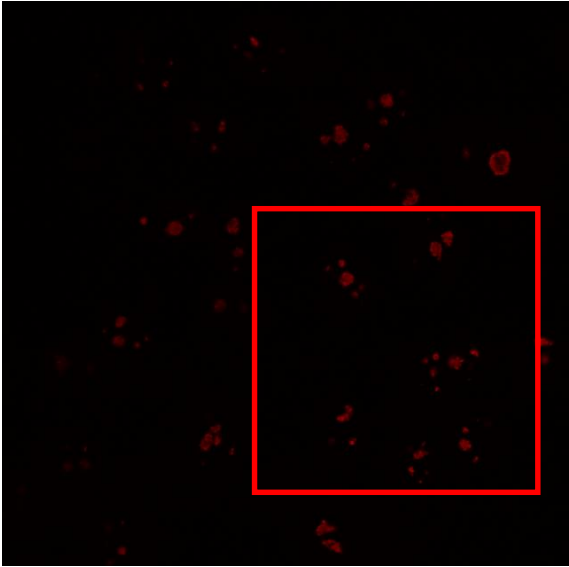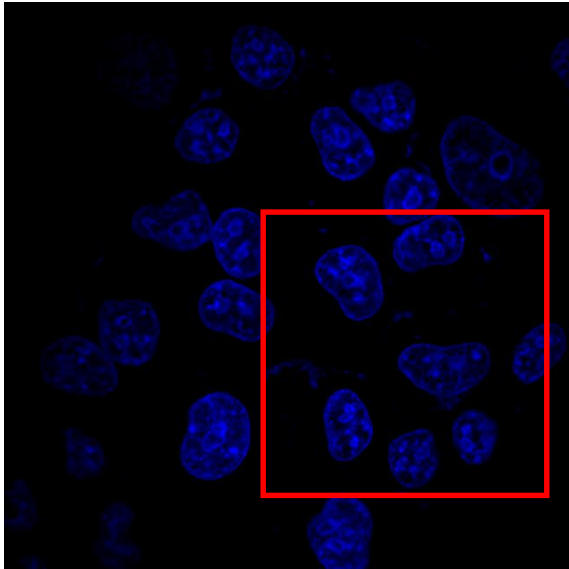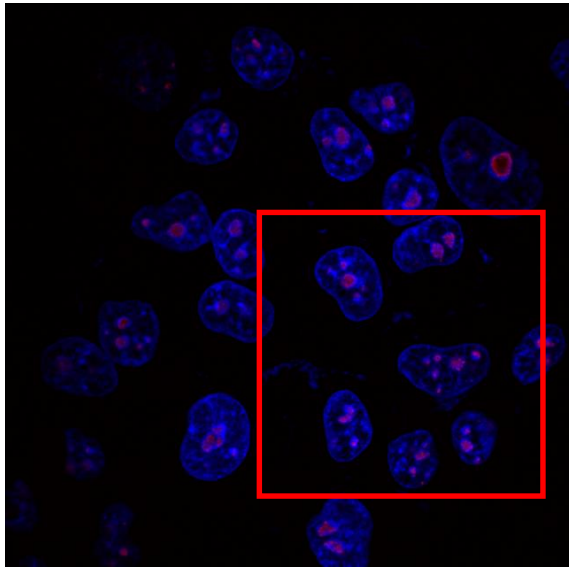

Appendix Figure S1B-right

shNC

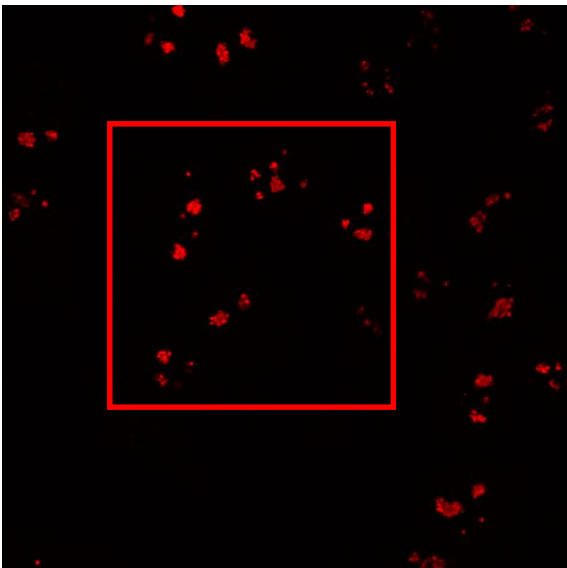

shNF90#1

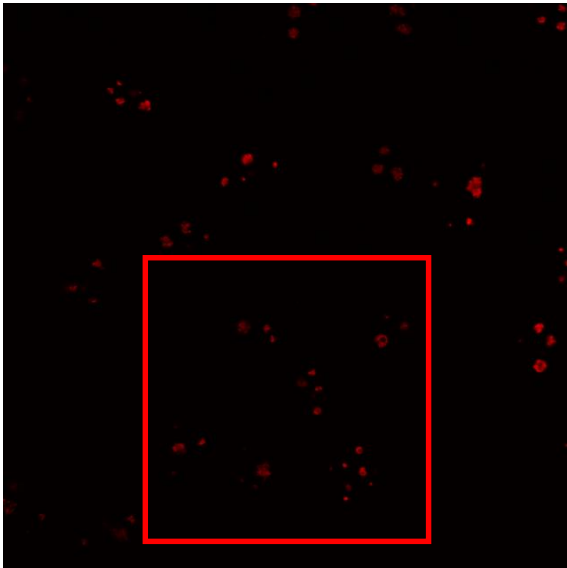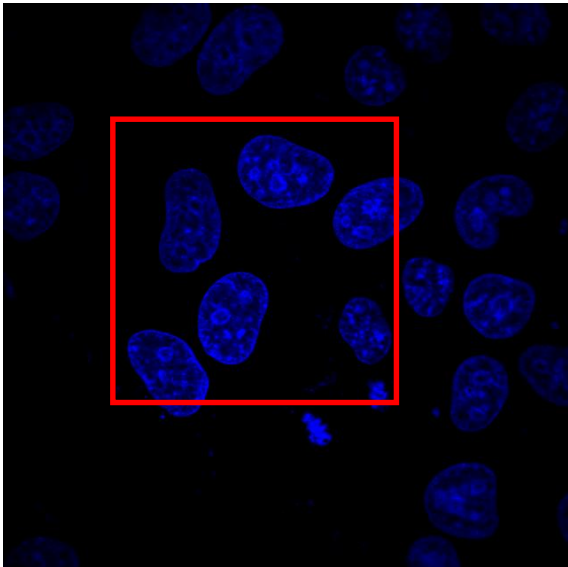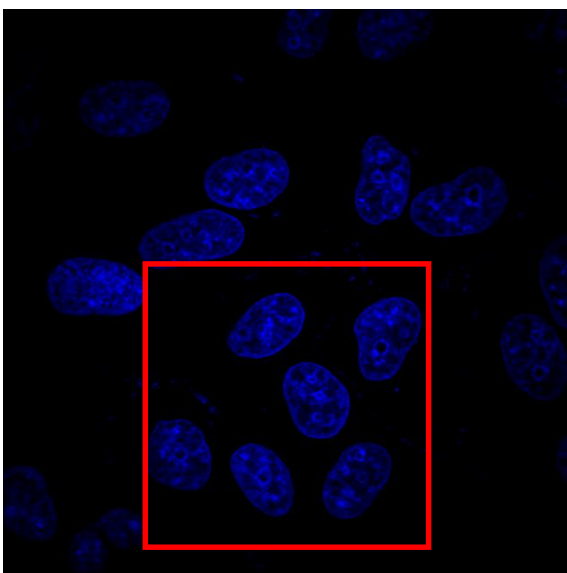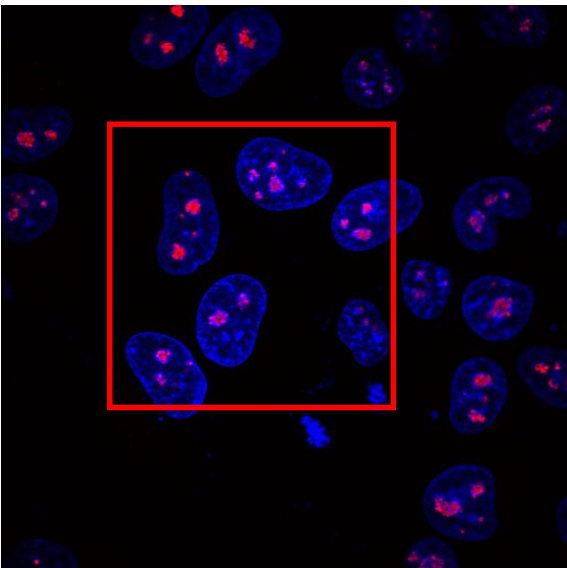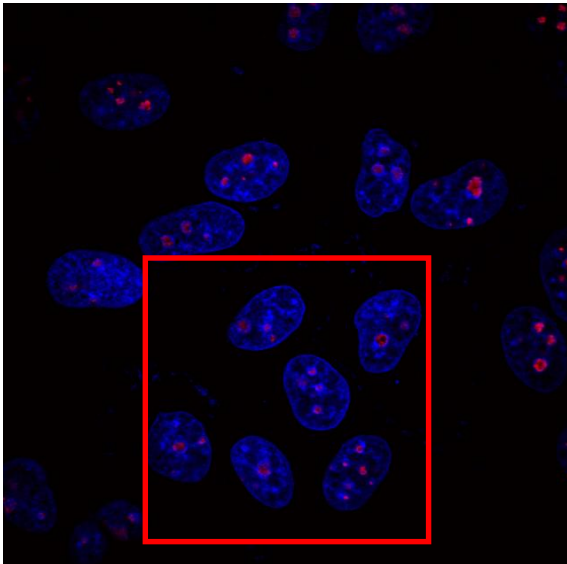

shNF90#2

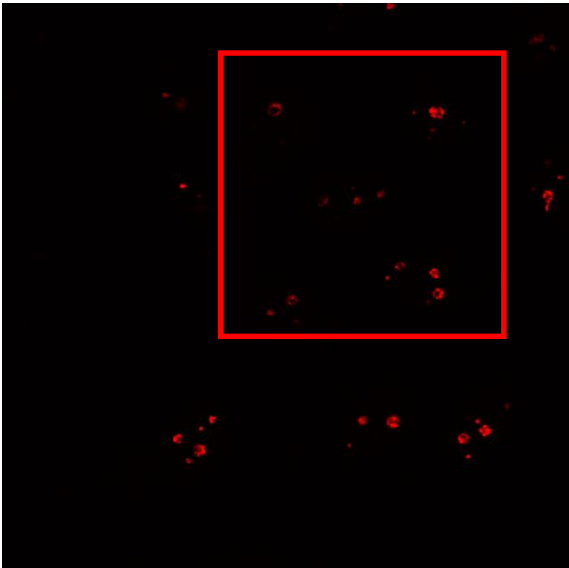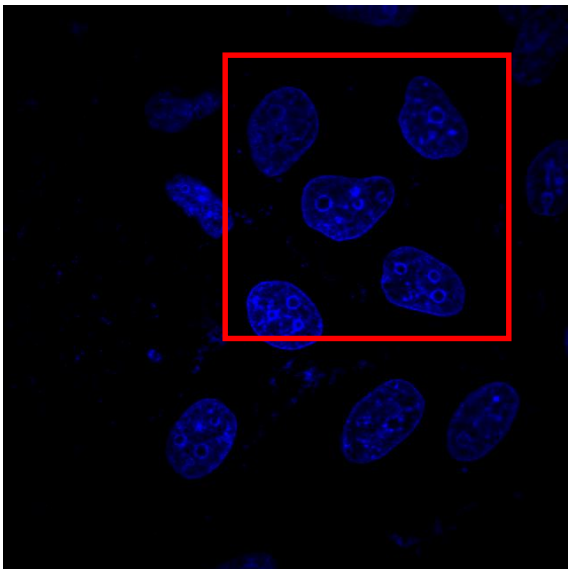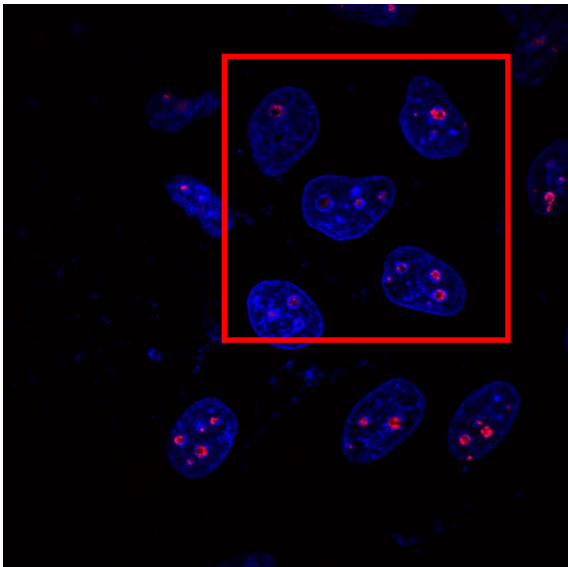

Supplement: Supplementary file 2 — Source Data for Appendix [file EMMM-13-e12834-s008.zip › Appendix_source_data/Source Data For Appendix Figure S1.pdf]

Appendix Figure S4A-left

Mock

FK506

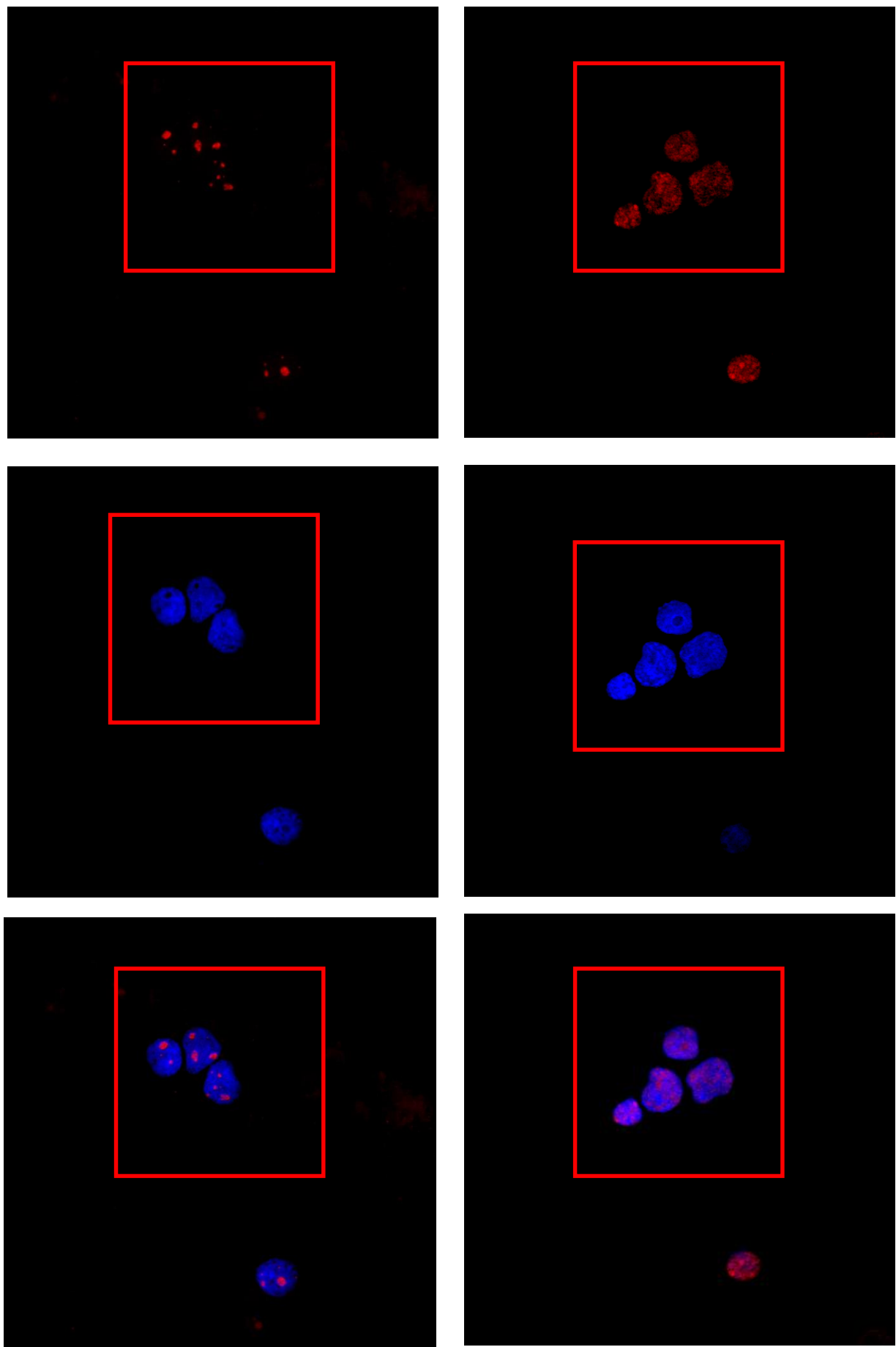

Appendix Figure S4A-right

Mock

FK506

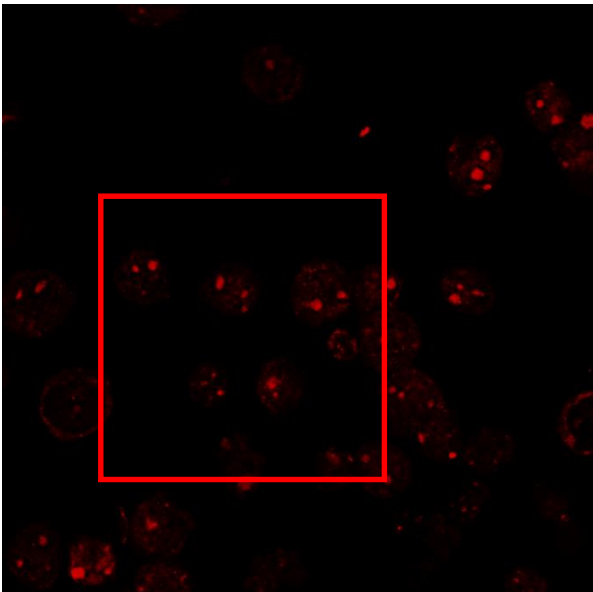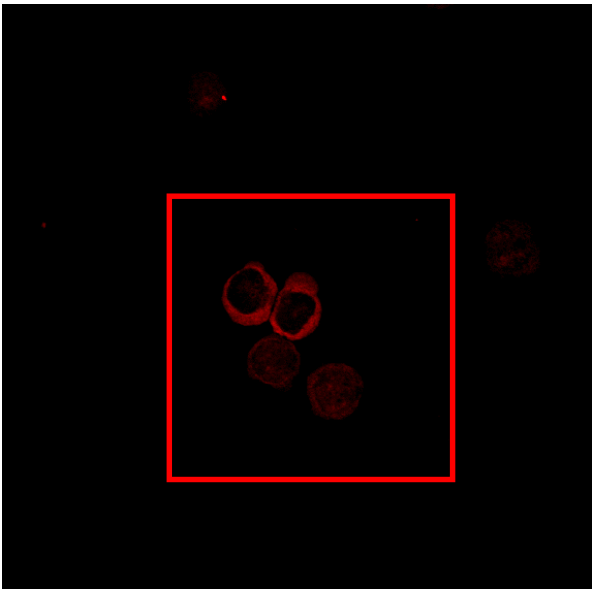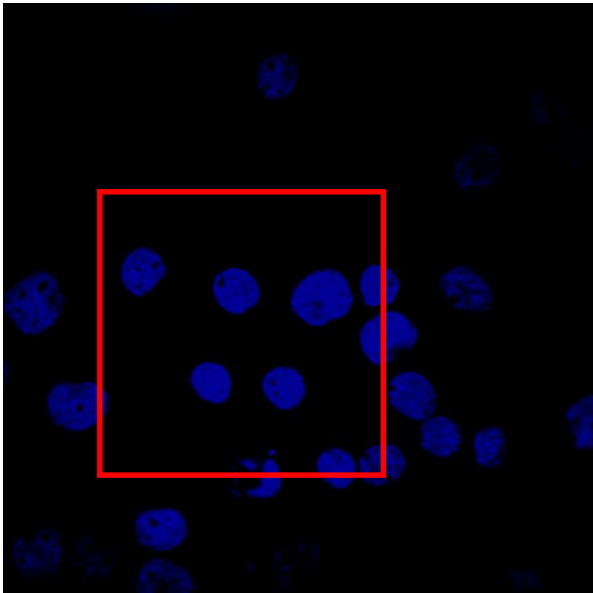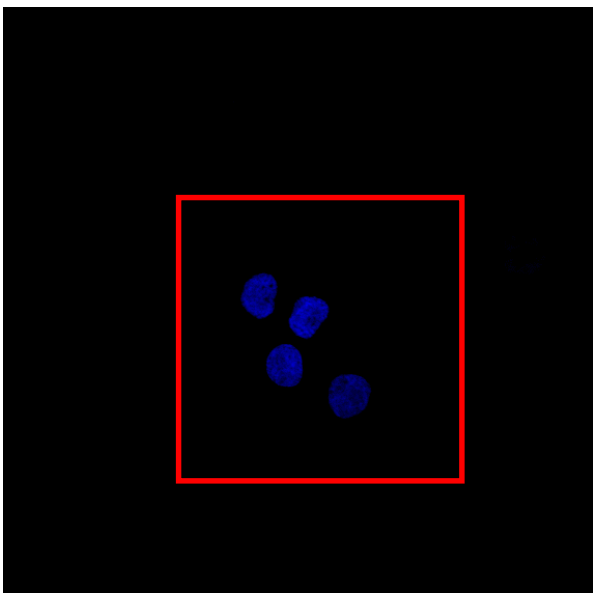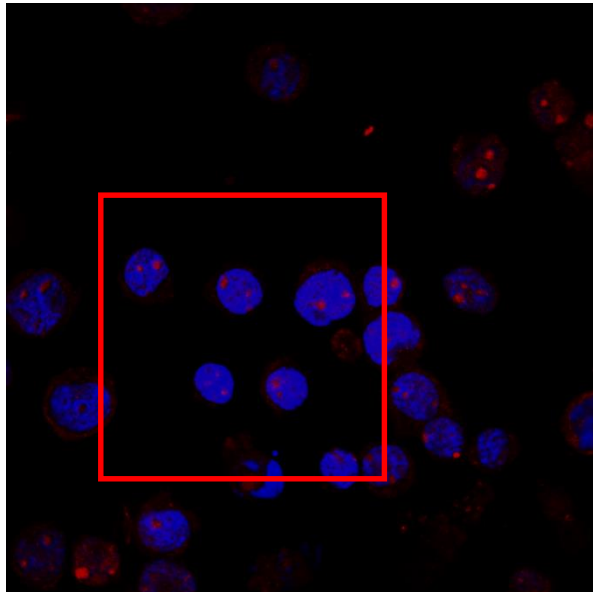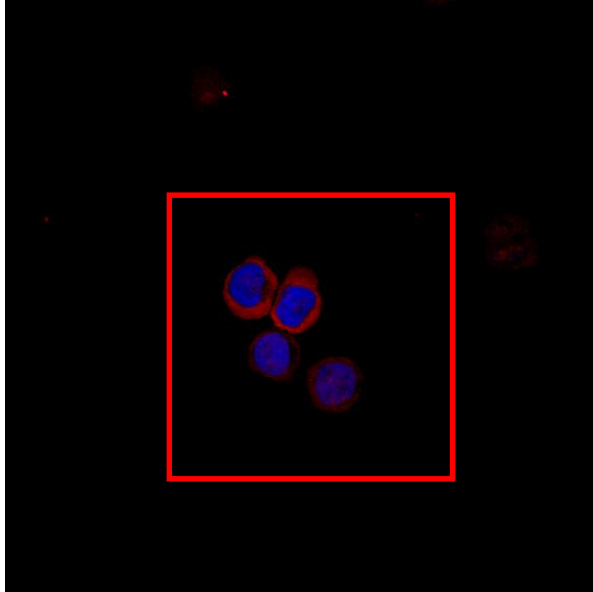

Supplement: Supplementary file 2 — Source Data for Appendix [file EMMM-13-e12834-s008.zip › Appendix_source_data/Source Data For Appendix Figure S4A.pdf]

EMSA (figure 2D)

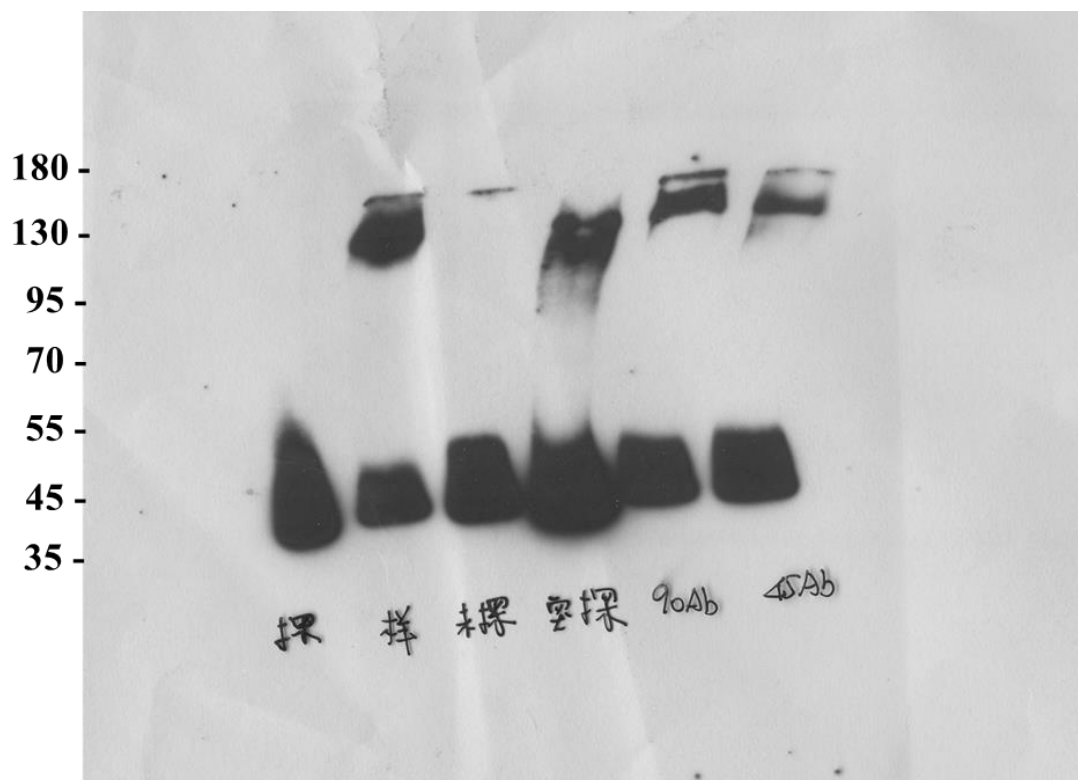

Supplement: Supplementary file 5 — Source Data for Figure 2 [file EMMM-13-e12834-s003.zip › Fig_2_source_data/Source Data For Figure 2D-.pdf]

## EMSA (figure 2D)

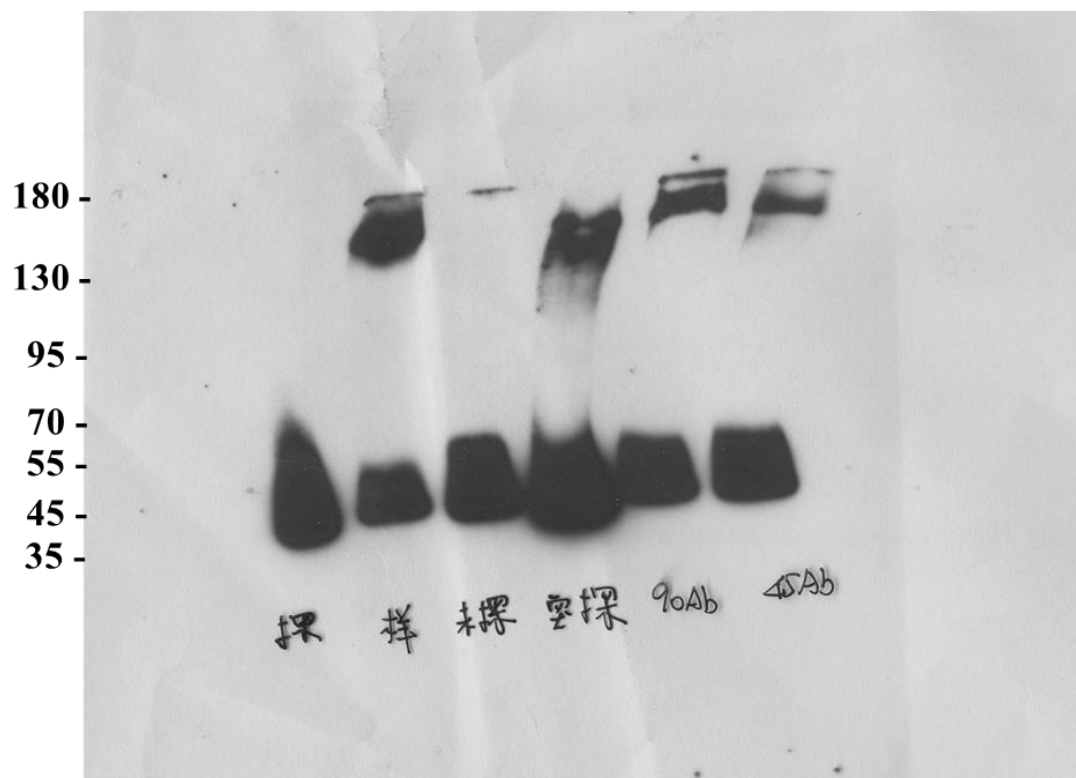

Supplement: Supplementary file 5 — Source Data for Figure 2 [file EMMM-13-e12834-s003.zip › Fig_2_source_data/Source Data For Figure 2D.pdf]

**Figure 5E**

**Mock**

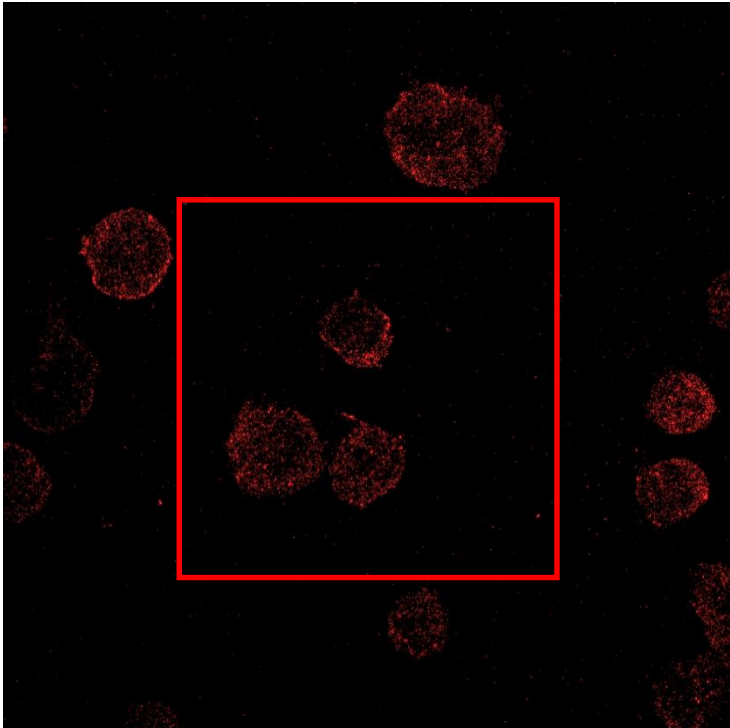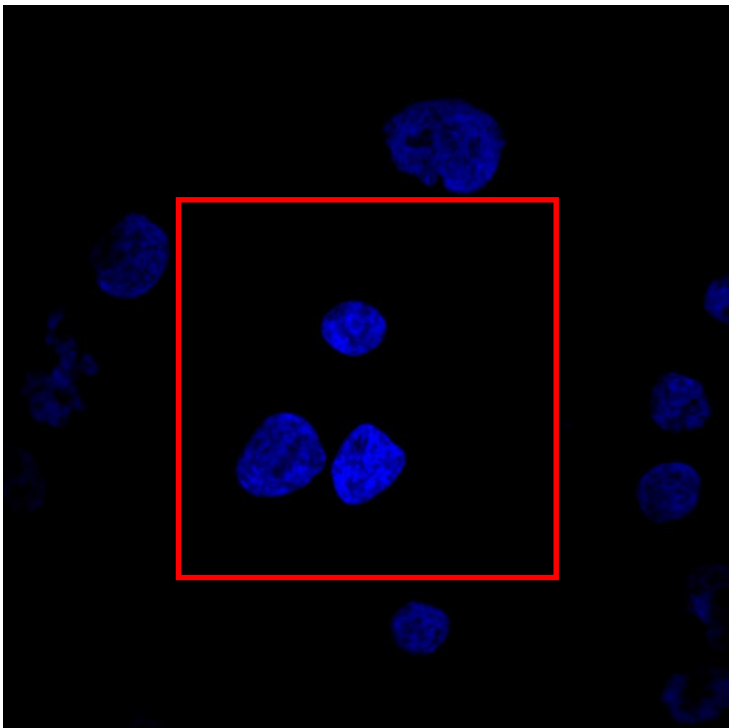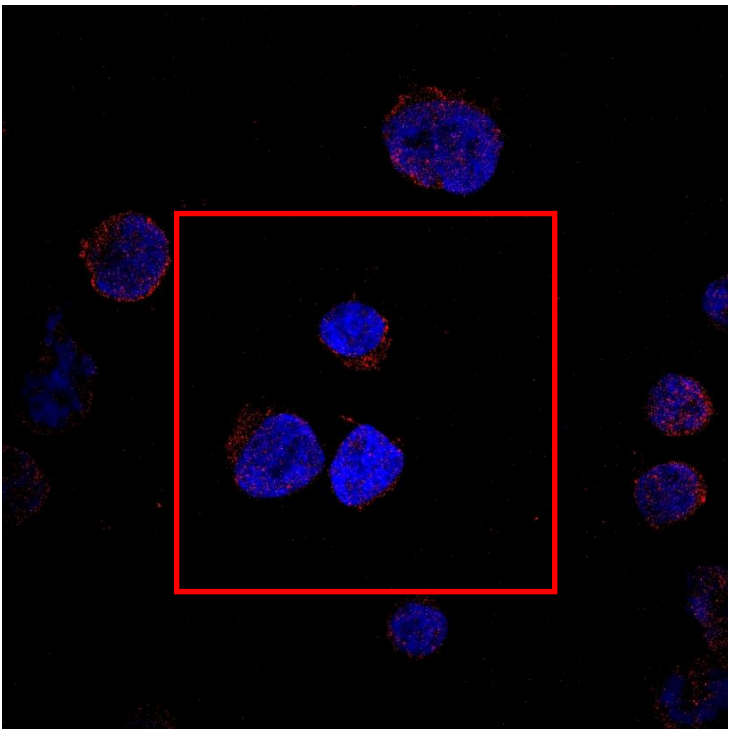

PI

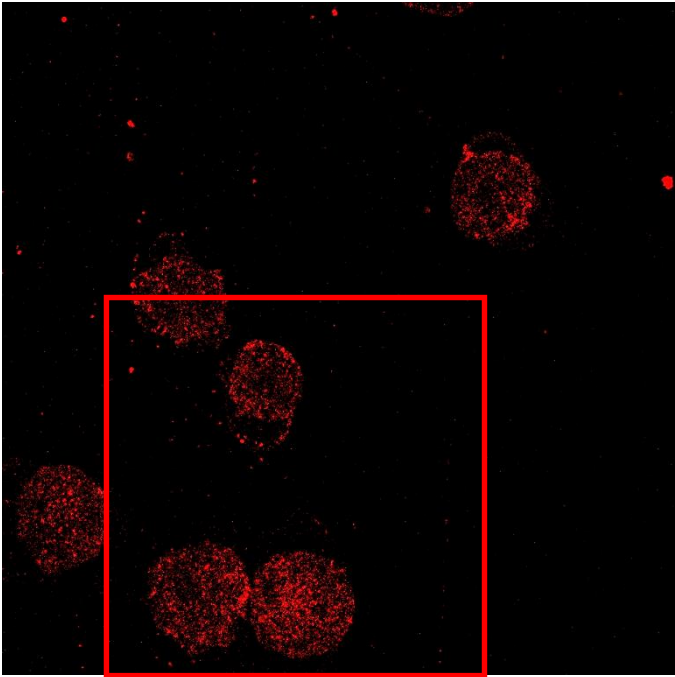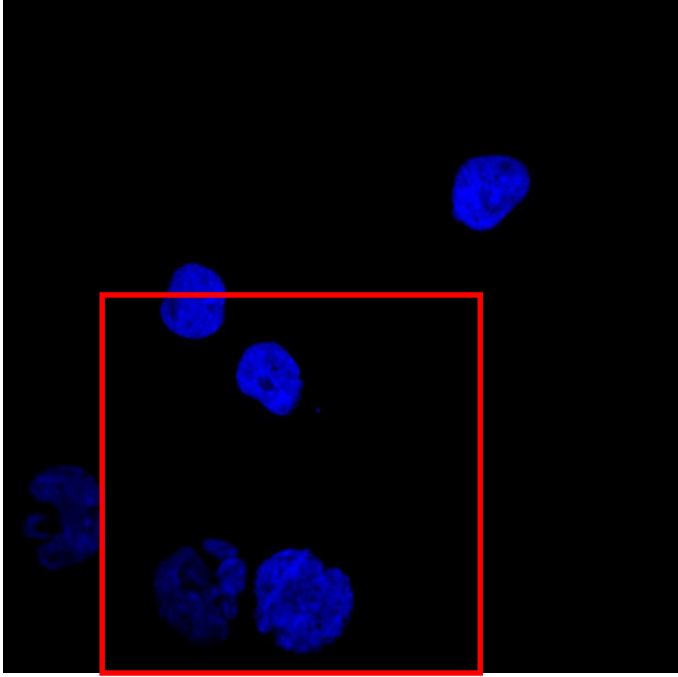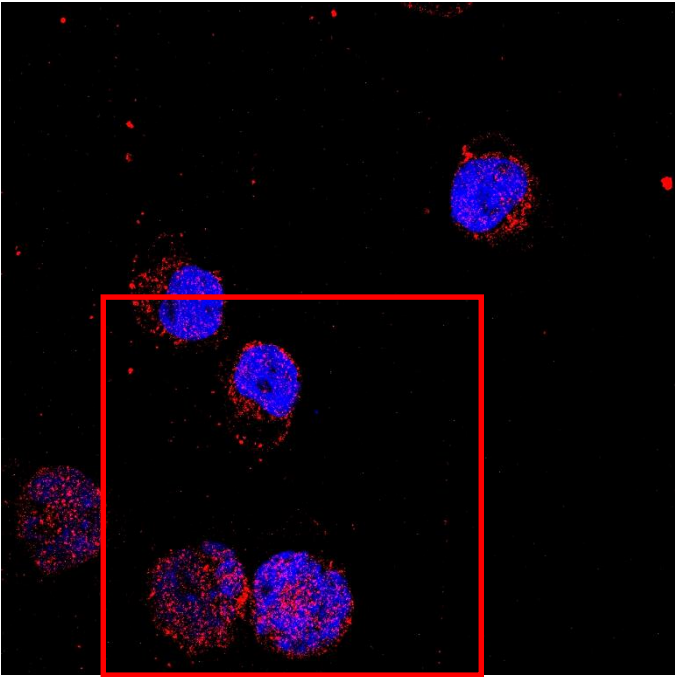

**PI+FK506**

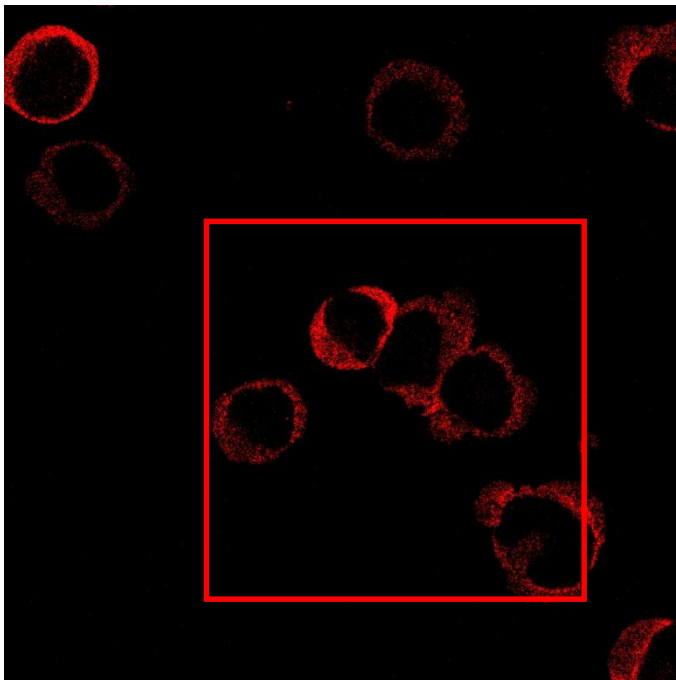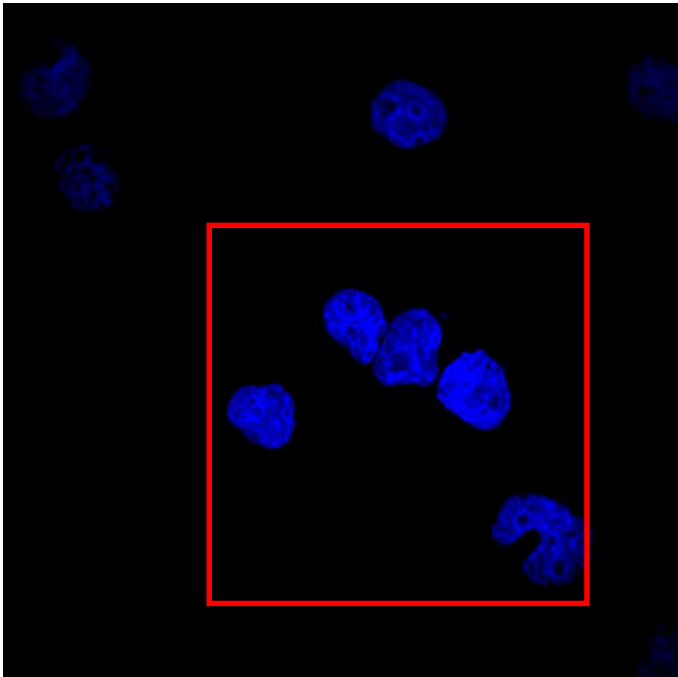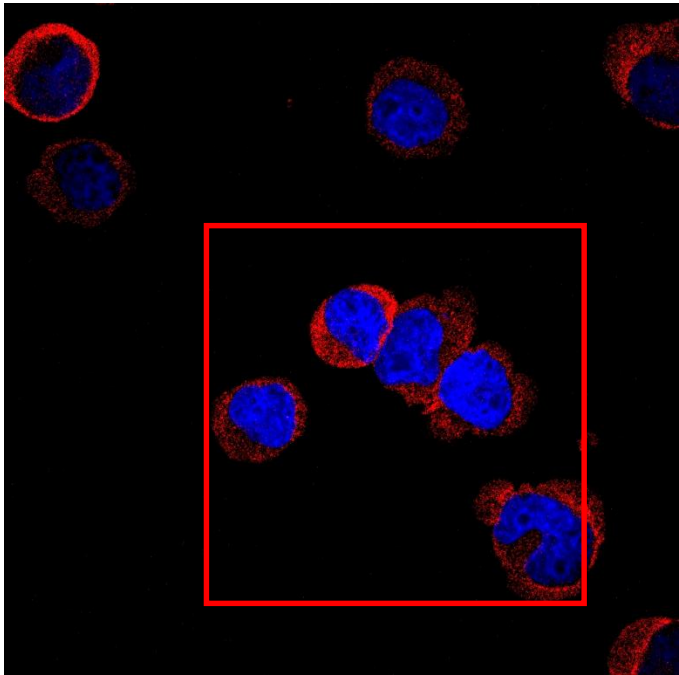

PI+CX5461

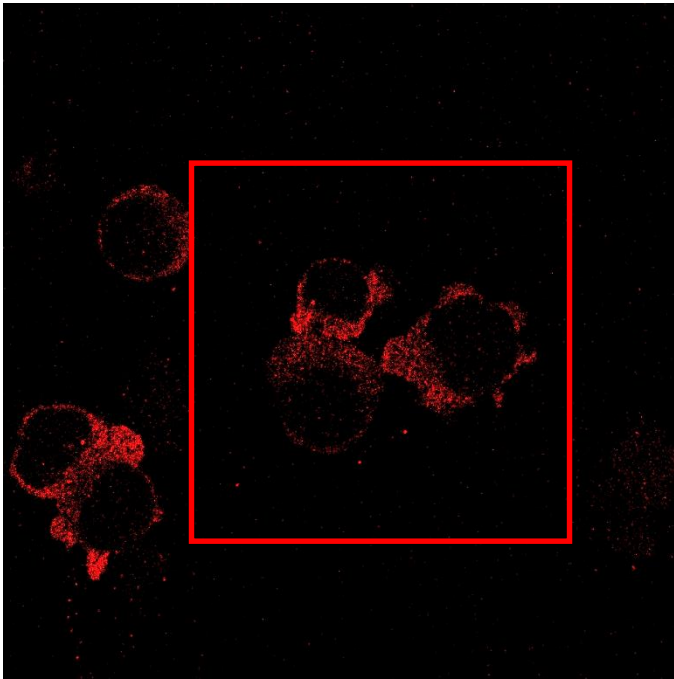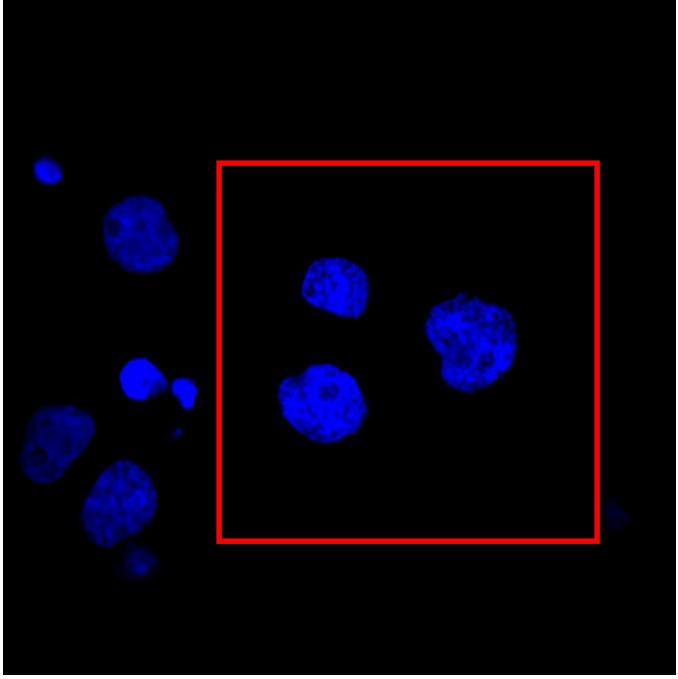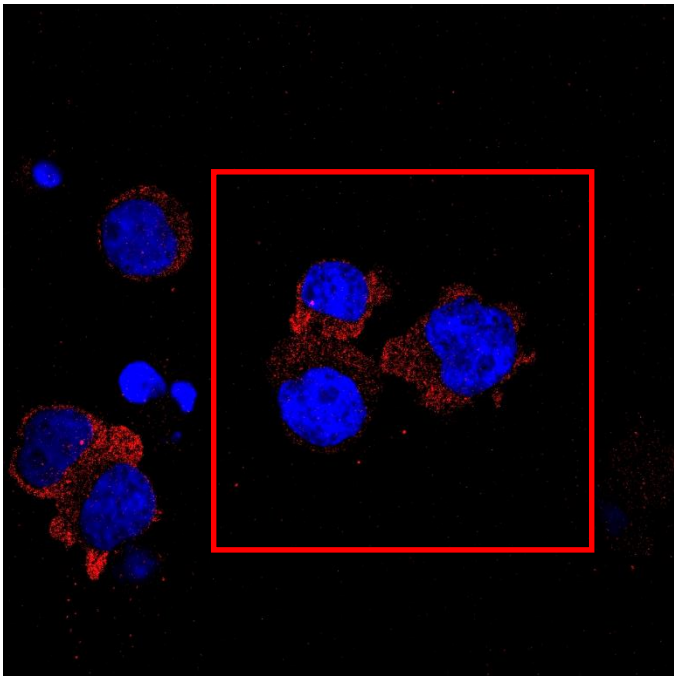

Supplement: Supplementary file 8 — Source Data for Figure 5 [file EMMM-13-e12834-s006.pdf]

**Figure 6E**

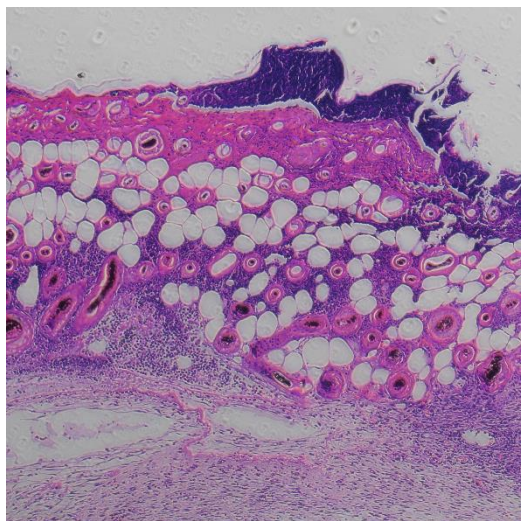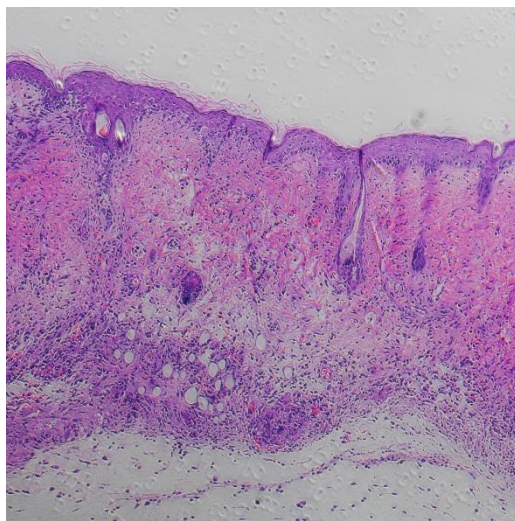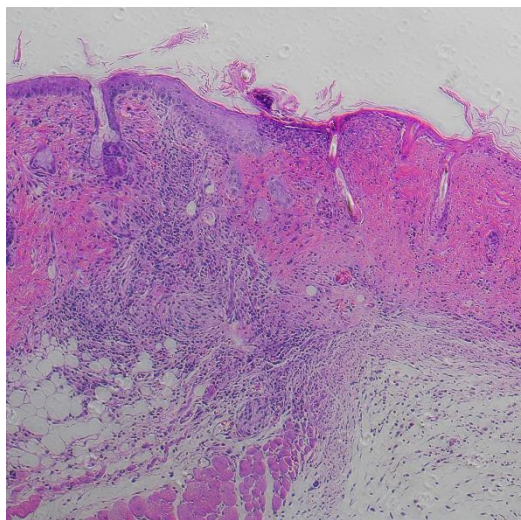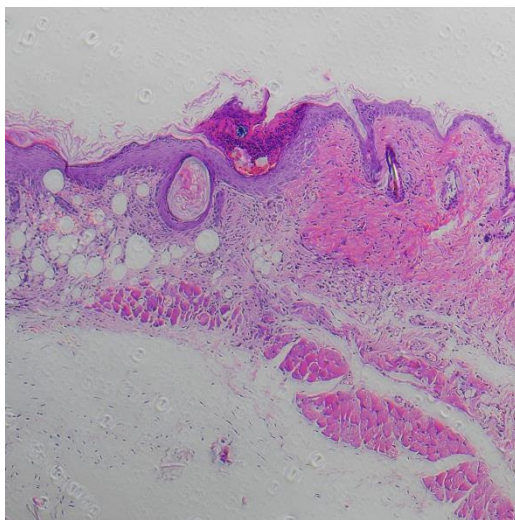

**Figure 6J**

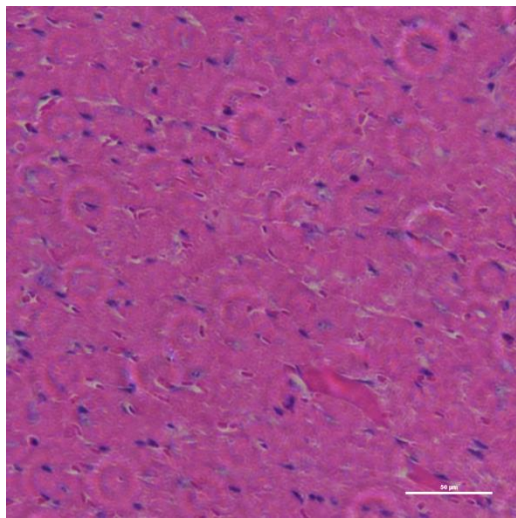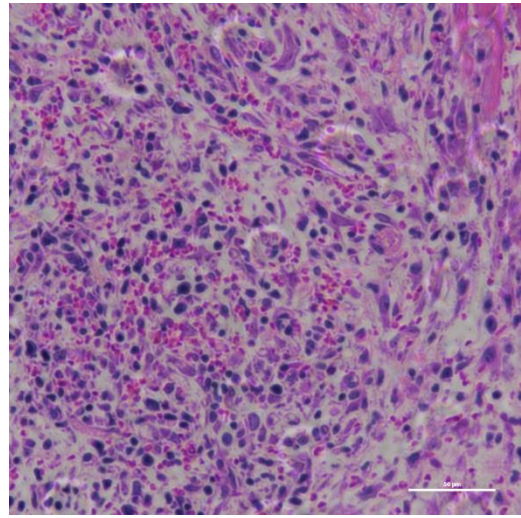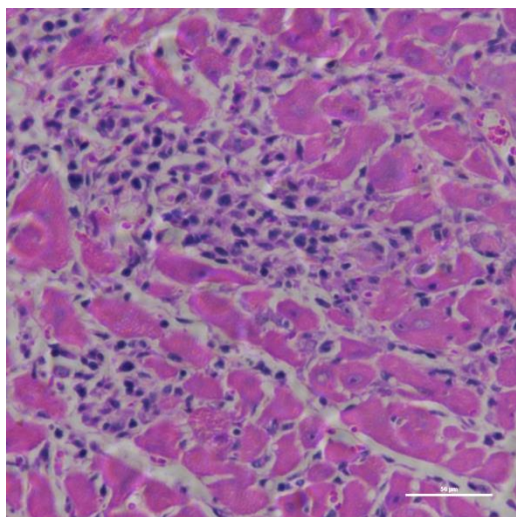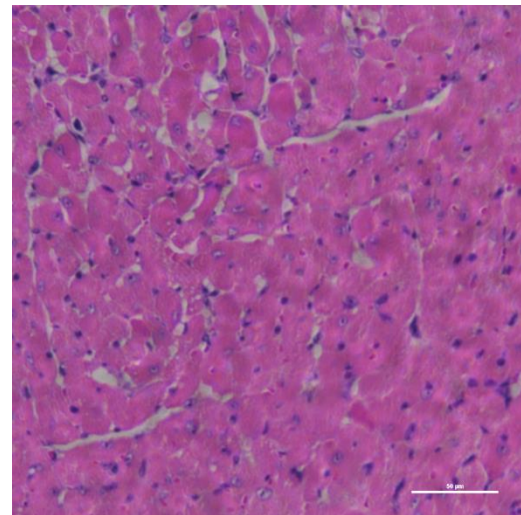

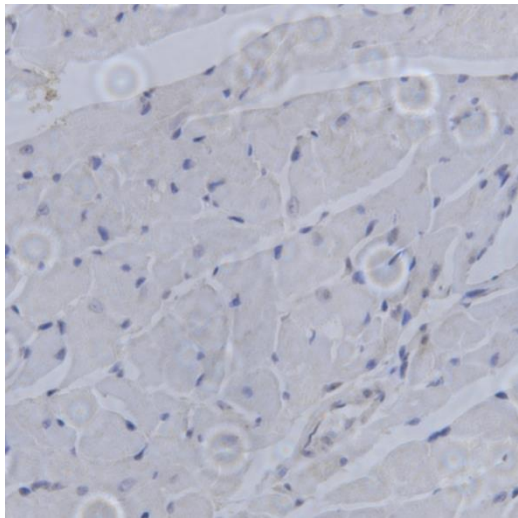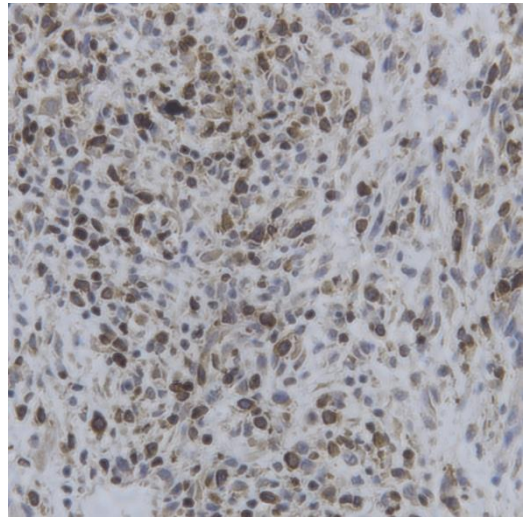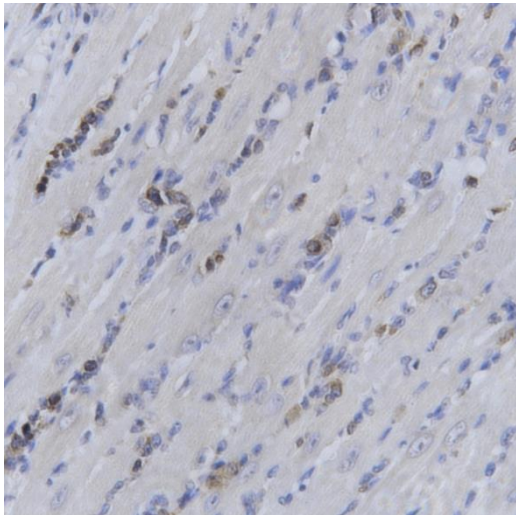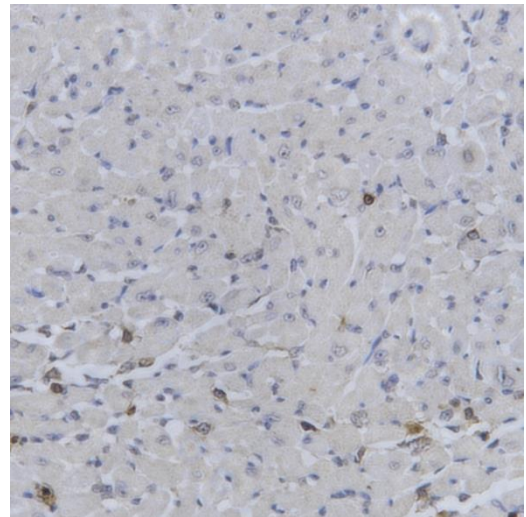

Supplement: Supplementary file 9 — Source Data for Figure 6 [file EMMM-13-e12834-s007.pdf]
