# Supplementary material for: NF45/NF90‐mediated rDNA transcription provides a novel target for immunosuppressant development
Source: EMBO Mol Med. 2021 Feb 8;13(3):e12834. doi: 10.15252/emmm.202012834 (PMC7933818; doi:10.15252/emmm.202012834)

Confocal images of NF45+fibrillarin (figure 1A)

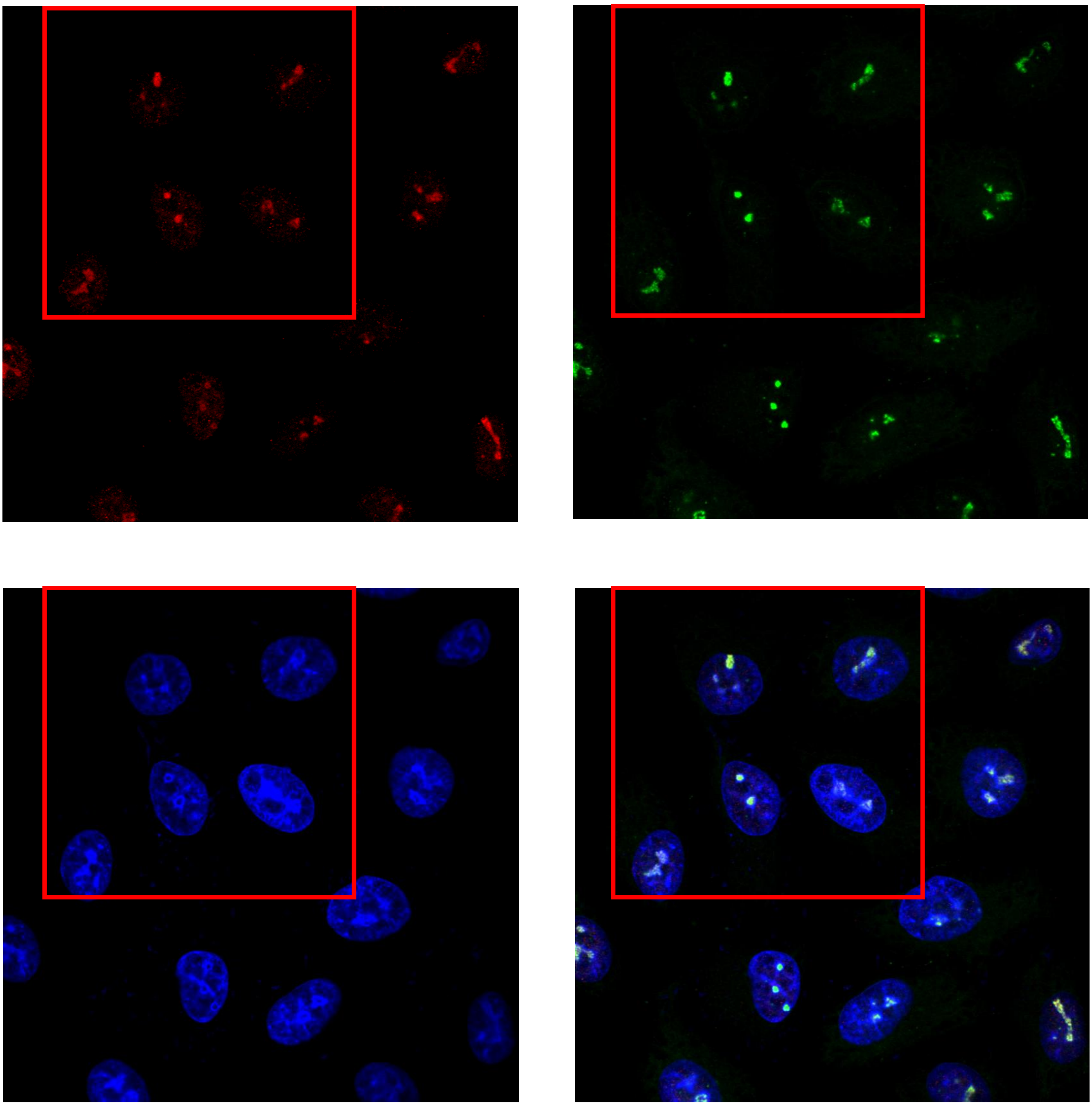

Confocal images of NF45+Nucleolin (figure 1A)

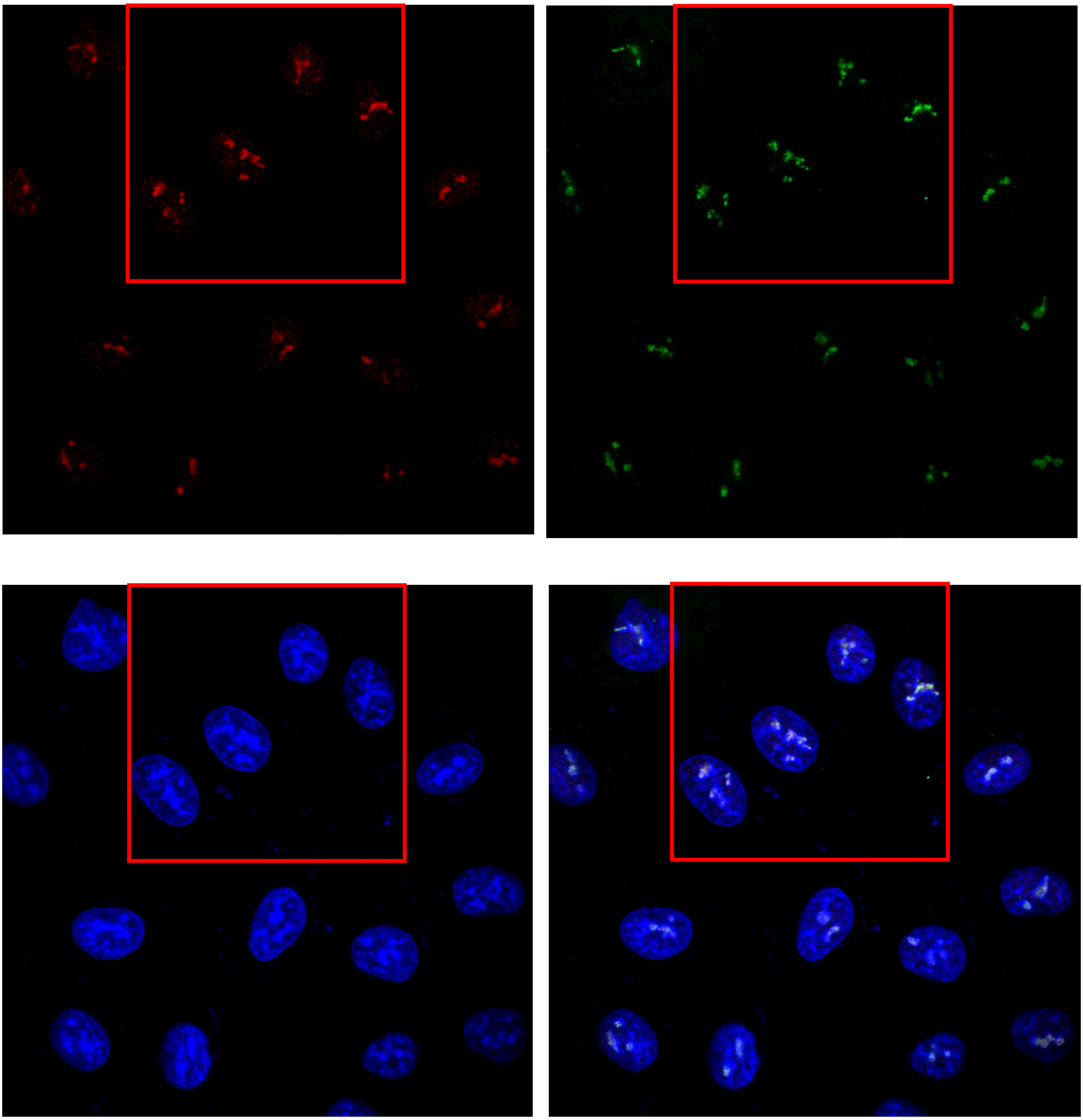

Confocal images of NF90+fibrillarin (figure 1A)

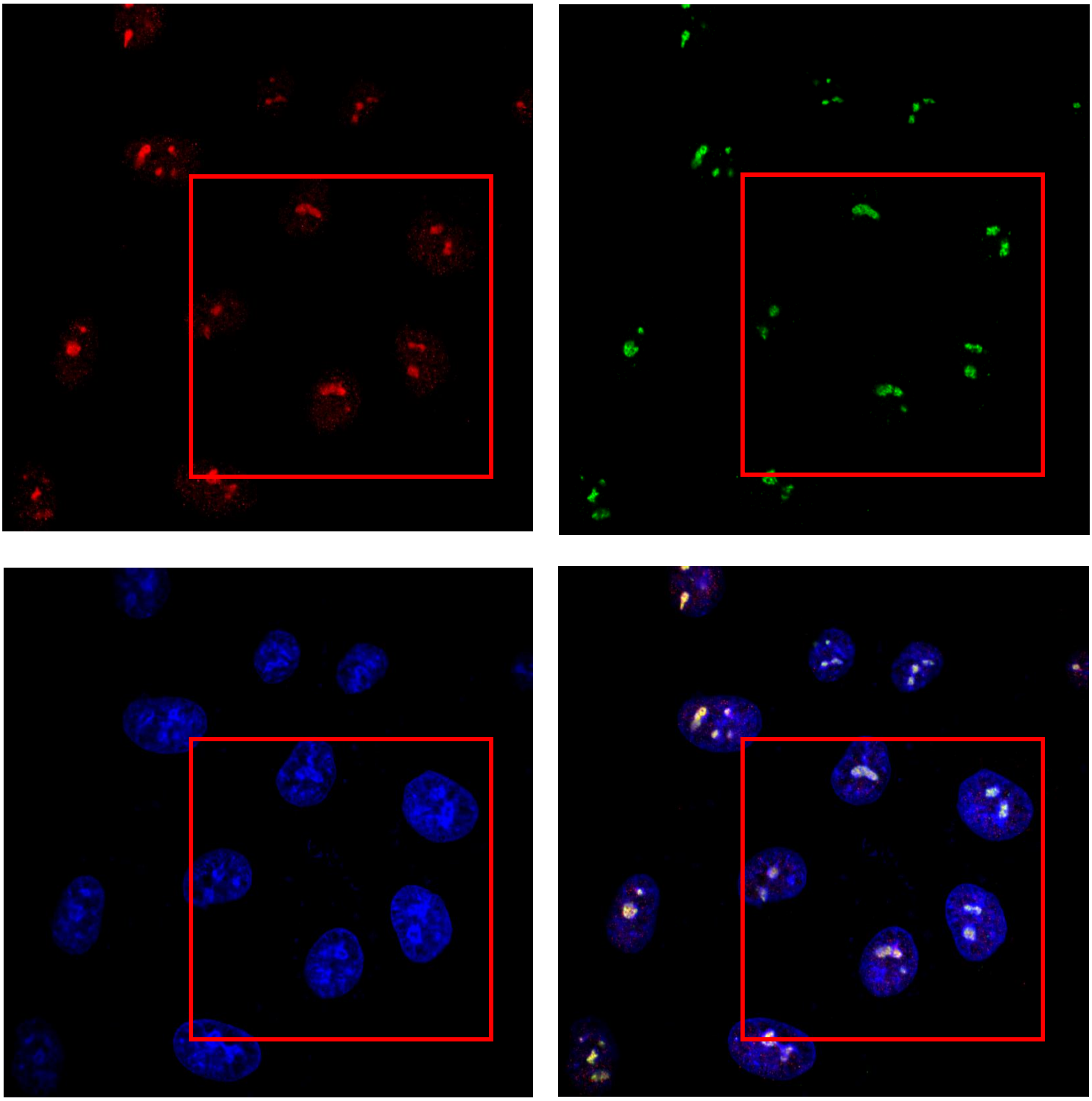

Confocal images of NF90+Nucleolin (figure 1A)

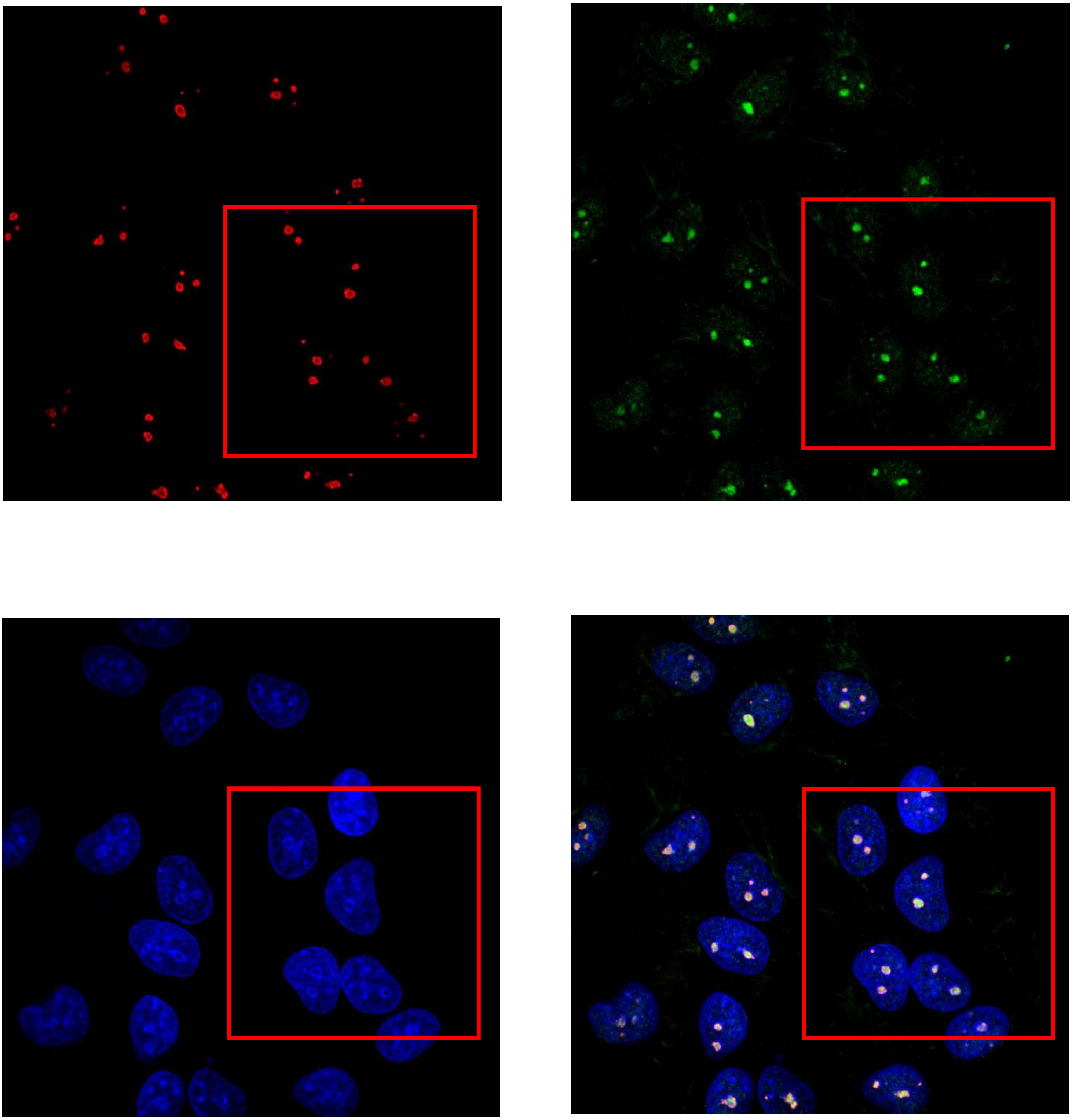

NF45 (figure 1E)

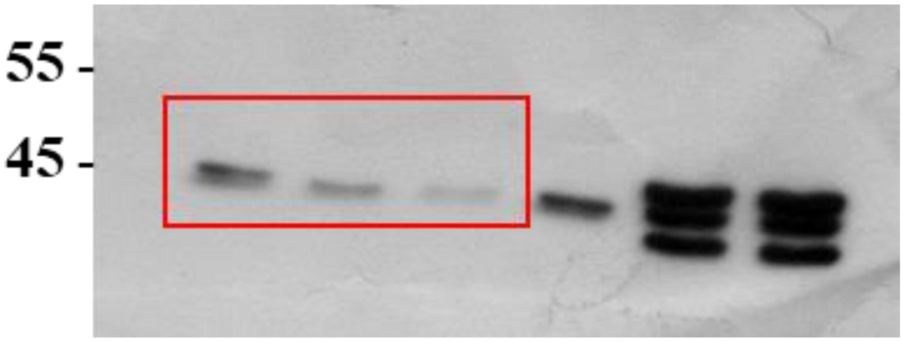

NF90 (figure 1E)

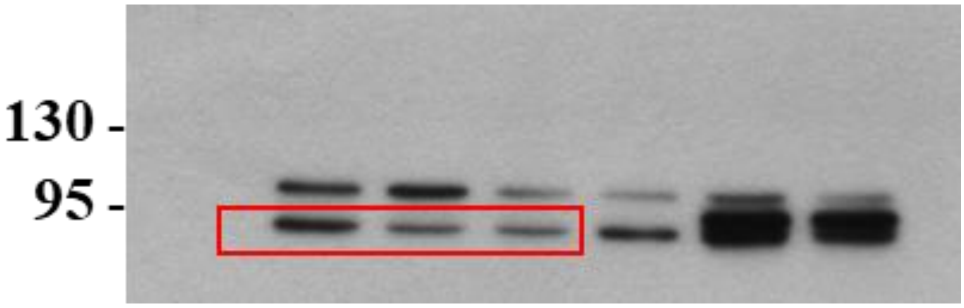

Supplement: Supplementary file 4 — Source Data for Figure 1 [file EMMM-13-e12834-s002.pdf]
