# Supplementary material for: NF45/NF90‐mediated rDNA transcription provides a novel target for immunosuppressant development
Source: EMBO Mol Med. 2021 Feb 8;13(3):e12834. doi: 10.15252/emmm.202012834 (PMC7933818; doi:10.15252/emmm.202012834)

Figure 3B

IB:NF90

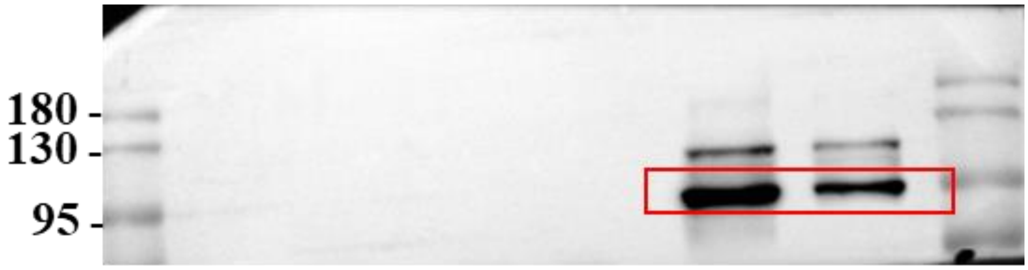

EGFP

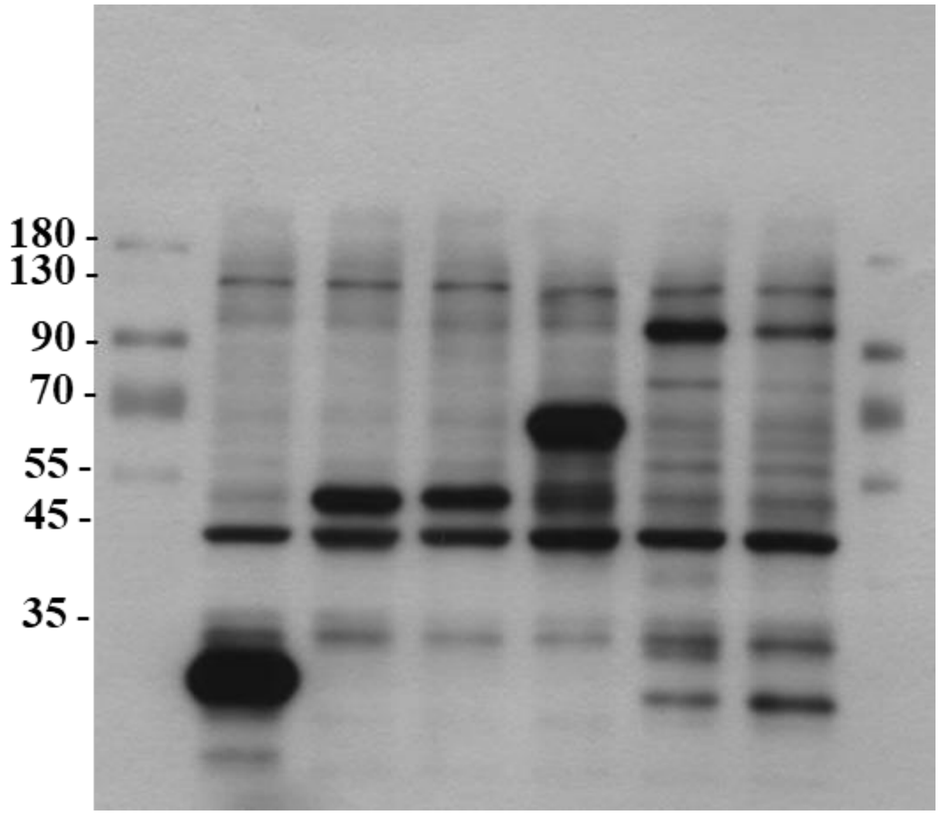

Figure 3C

Input:NF45

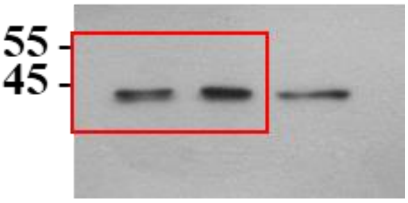

Input:NF90

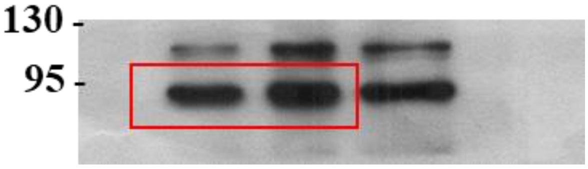

Input: UBF

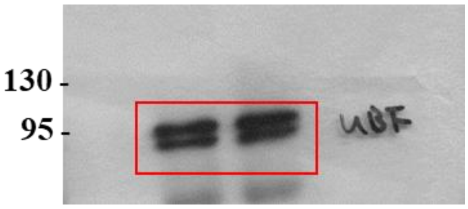

IP:NF45 IB:NF45

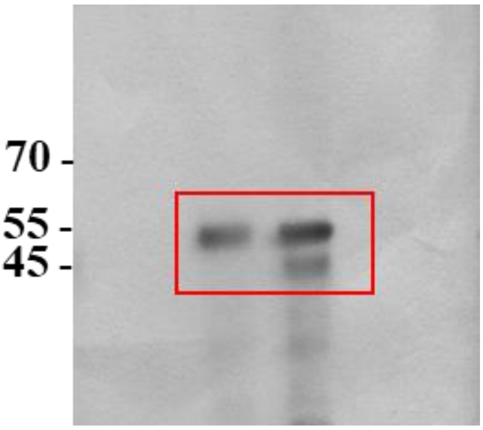

IP:NF45 IB:UBF

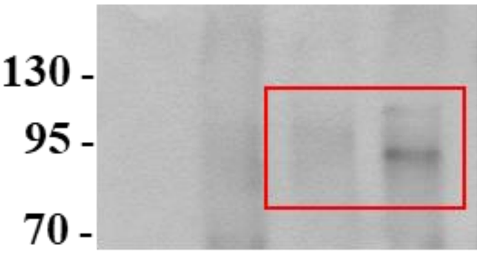

IP:NF90 IB:NF90

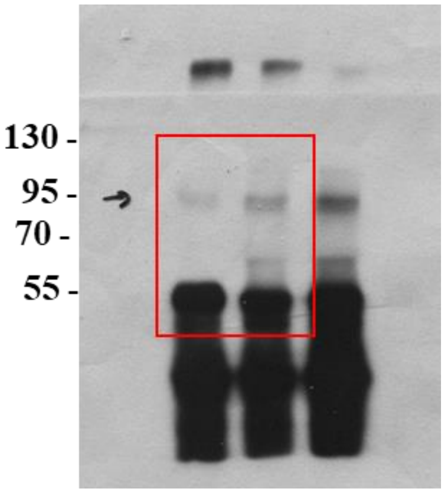

IP:NF90 IB:UBF

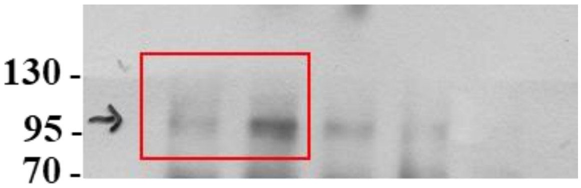

Figure 3D

For shNF45

RNA pol I

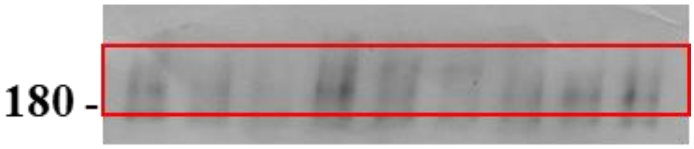

UBF

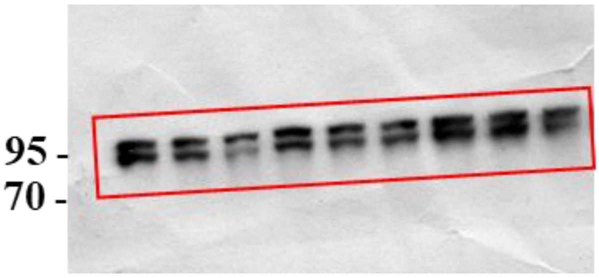

p-UBF

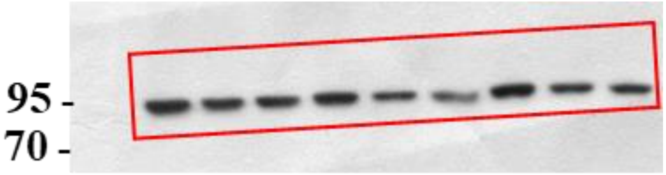

NF45

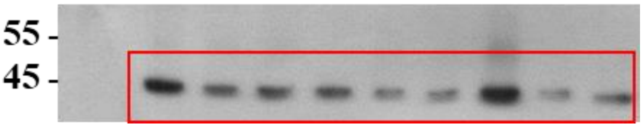

GAPDH

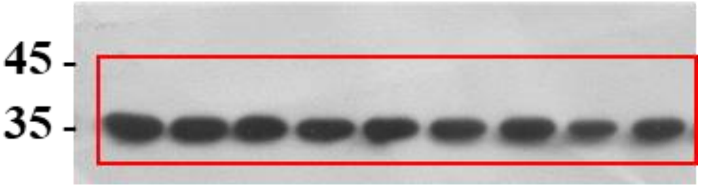

For shNF90

RNA pol I

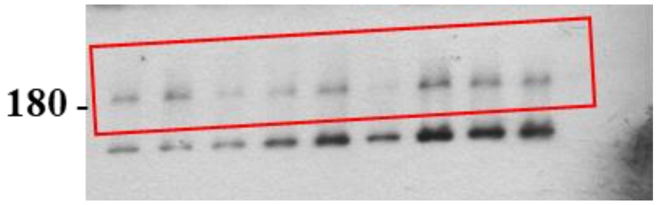

UBF

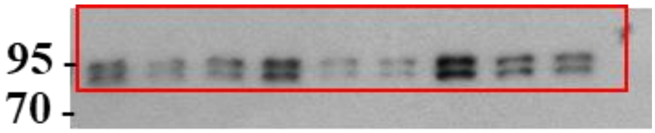

p-UBF

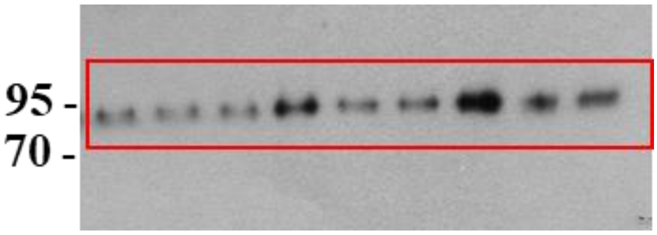

NF90

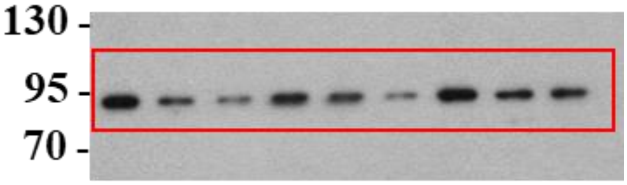

GAPDH

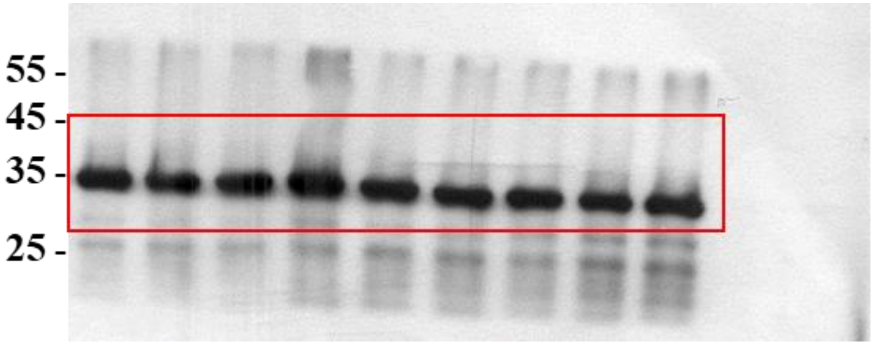

Figure 3G

NF45-Mock

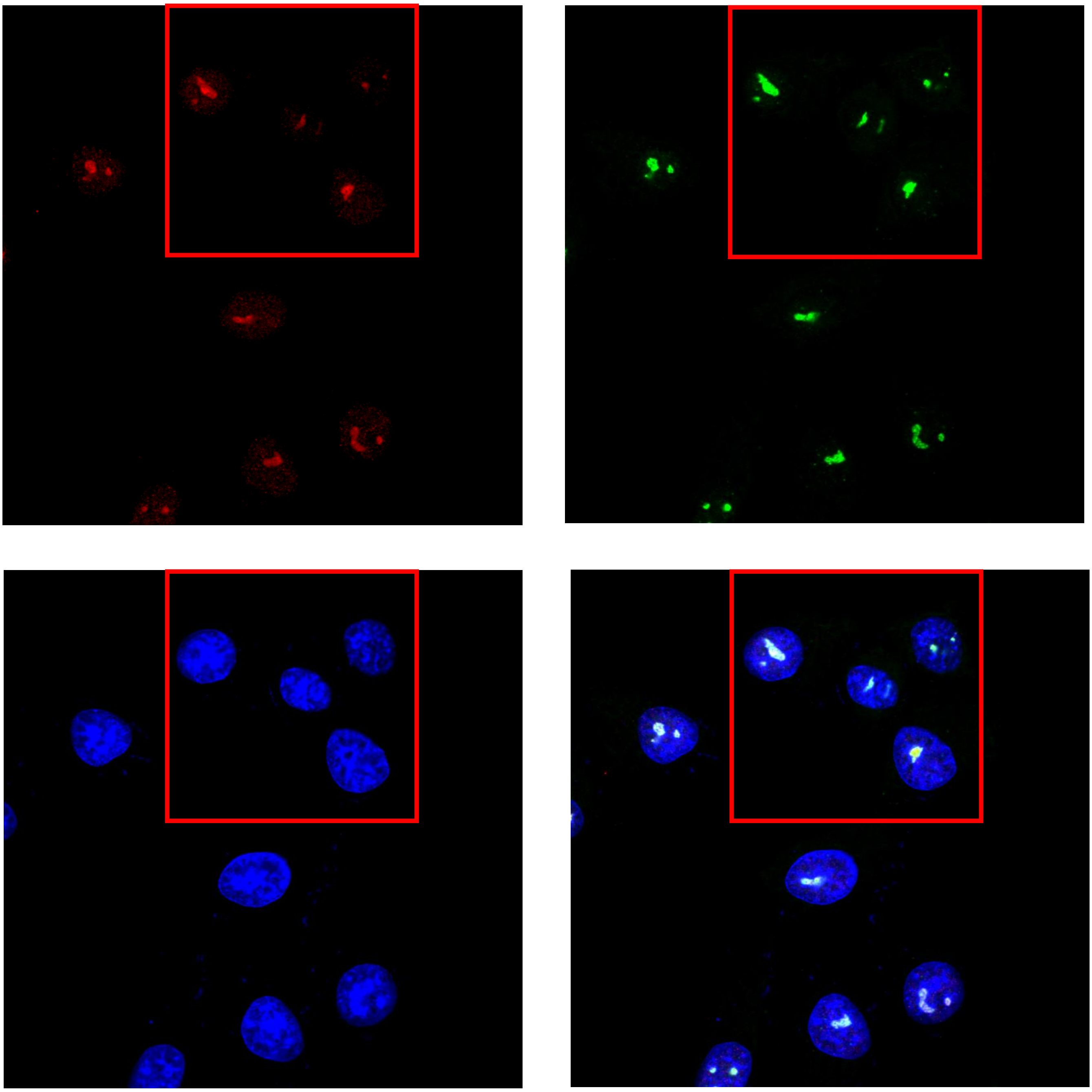

NF45-CX5461

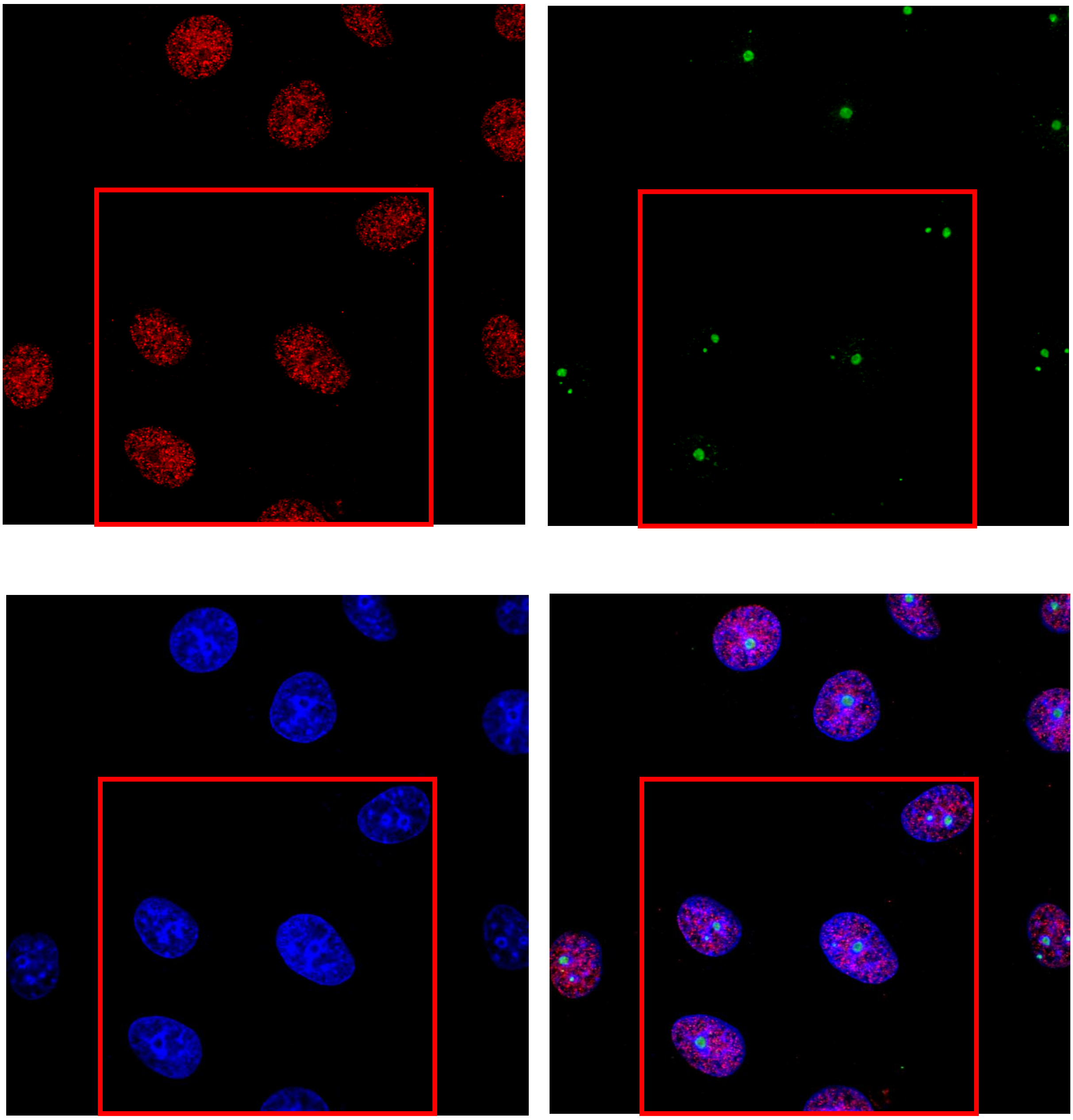

NF90-Mock

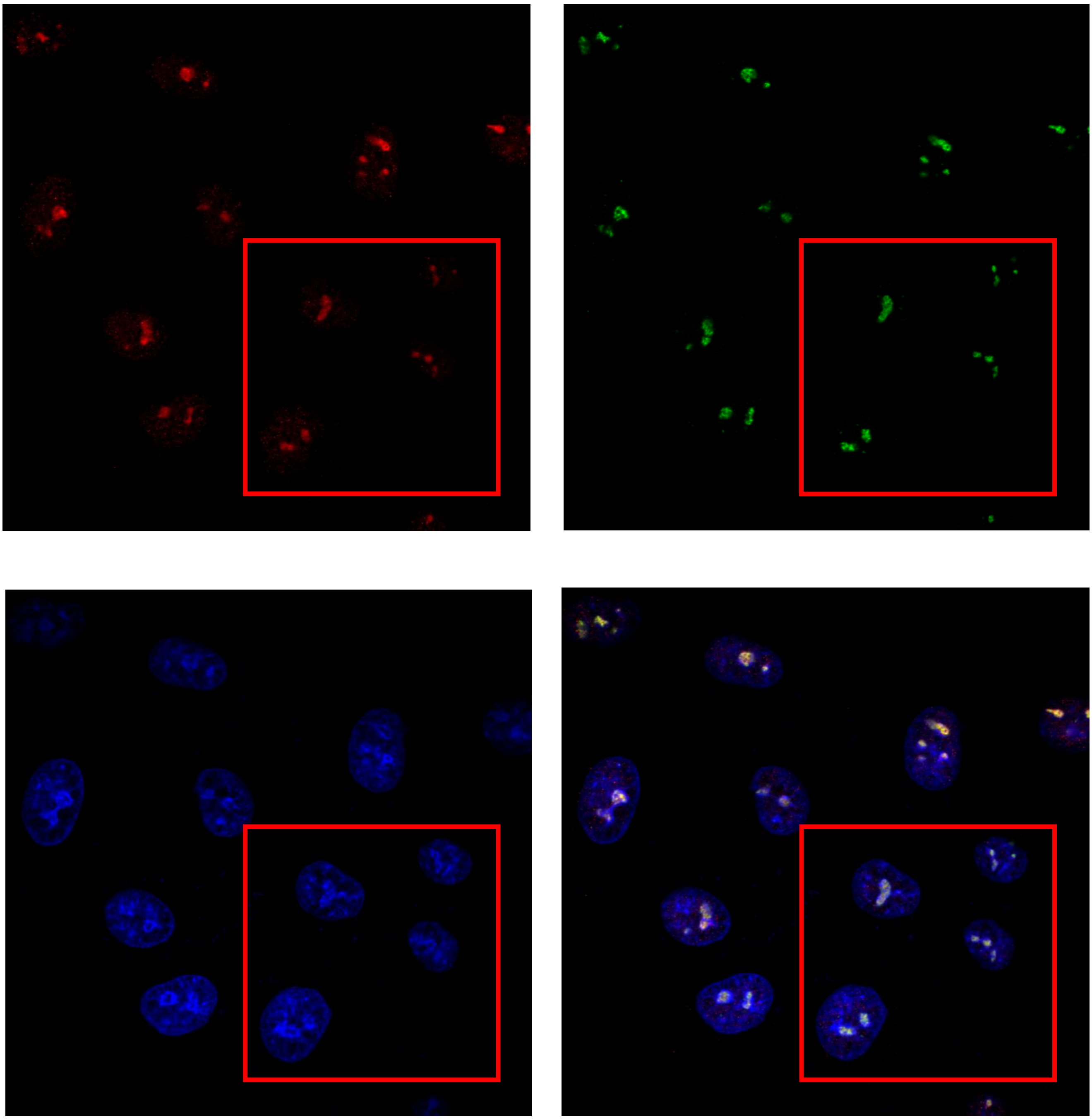

NF90-CX5461

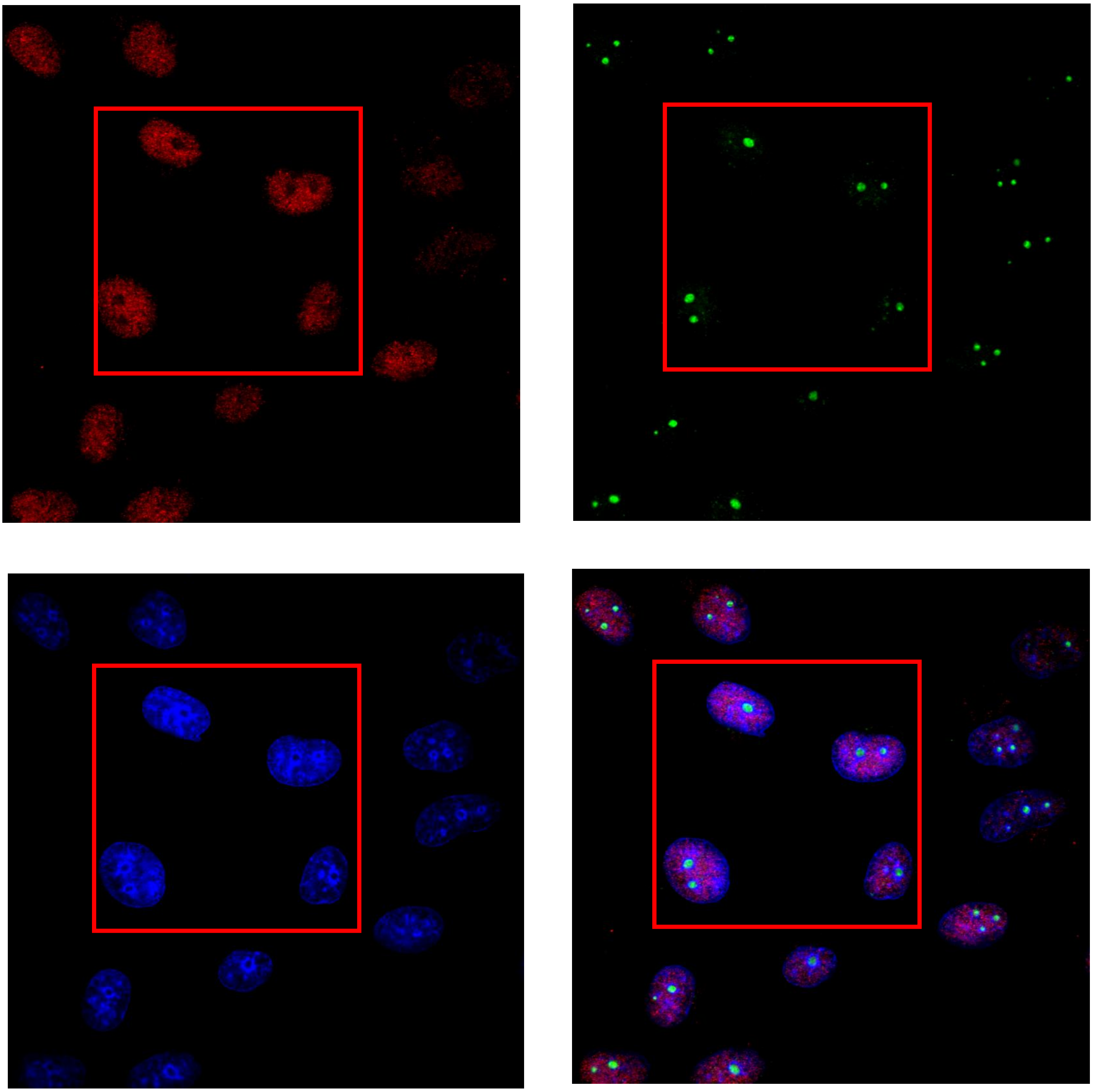

Supplement: Supplementary file 6 — Source Data for Figure 3 [file EMMM-13-e12834-s004.pdf]
