# Supplementary material for: NF45/NF90‐mediated rDNA transcription provides a novel target for immunosuppressant development
Source: EMBO Mol Med. 2021 Feb 8;13(3):e12834. doi: 10.15252/emmm.202012834 (PMC7933818; doi:10.15252/emmm.202012834)

## Figure 4E

## NF45-Mock

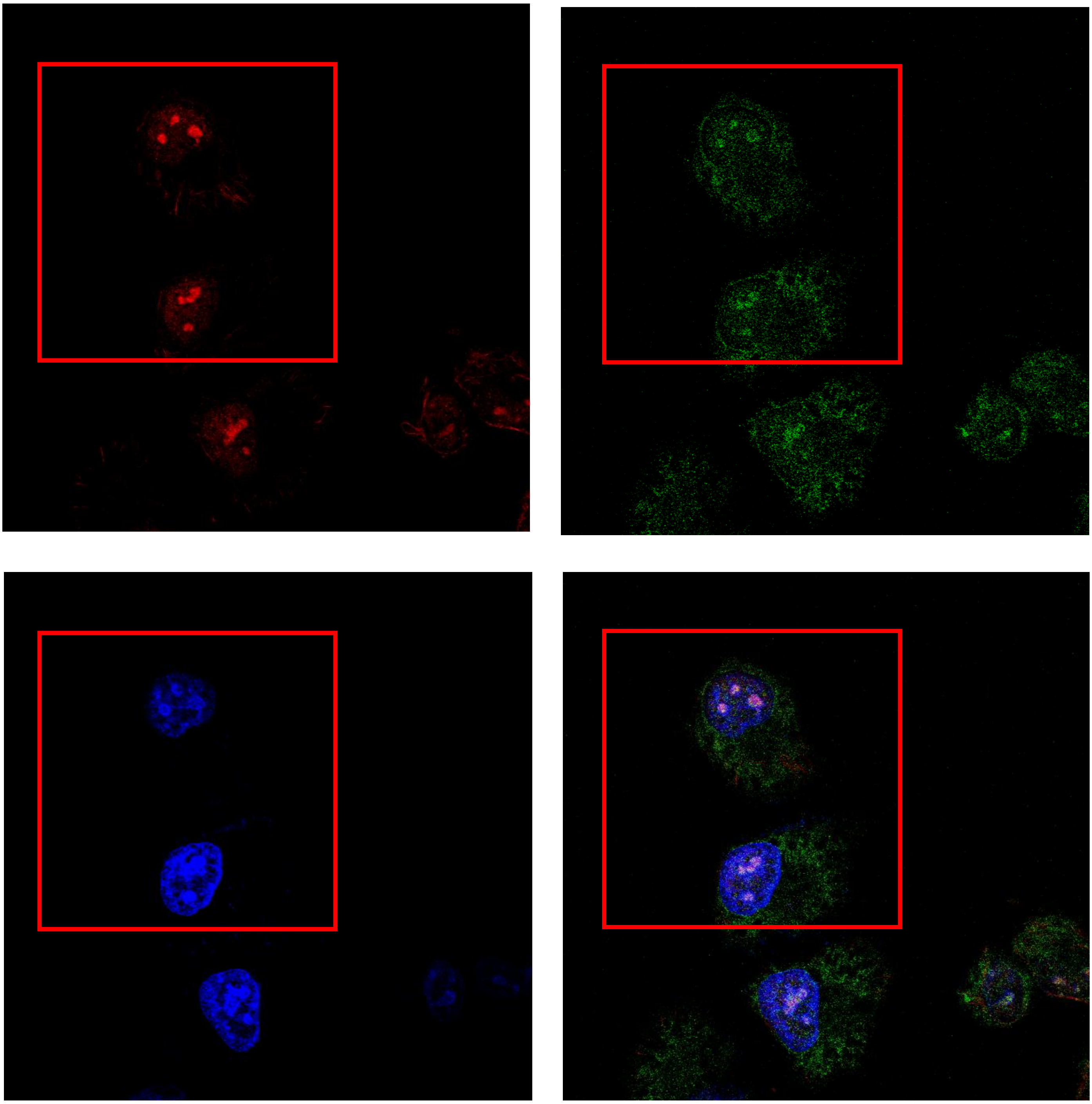

**NF45-PI**

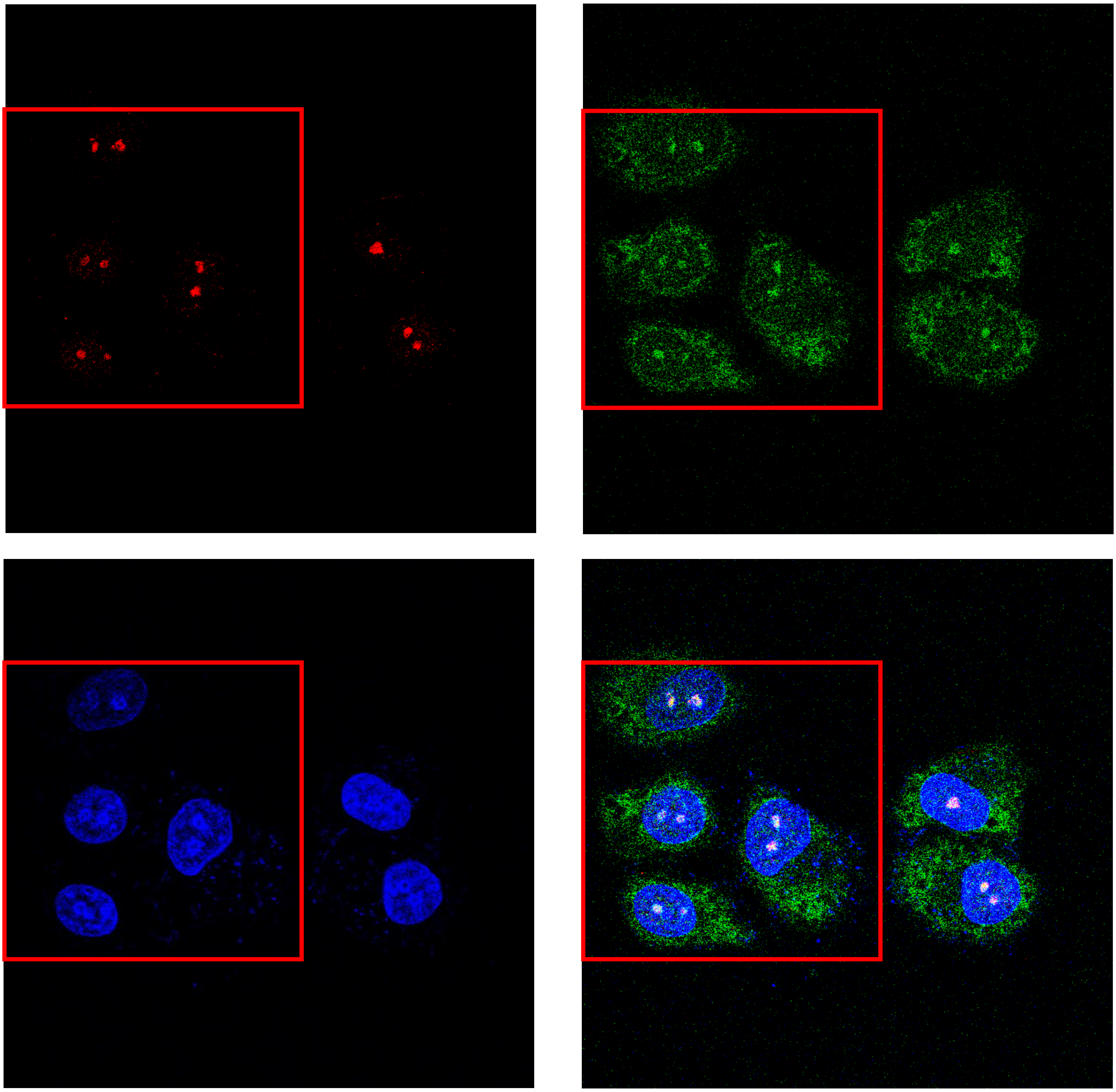

**NF90-Mock**

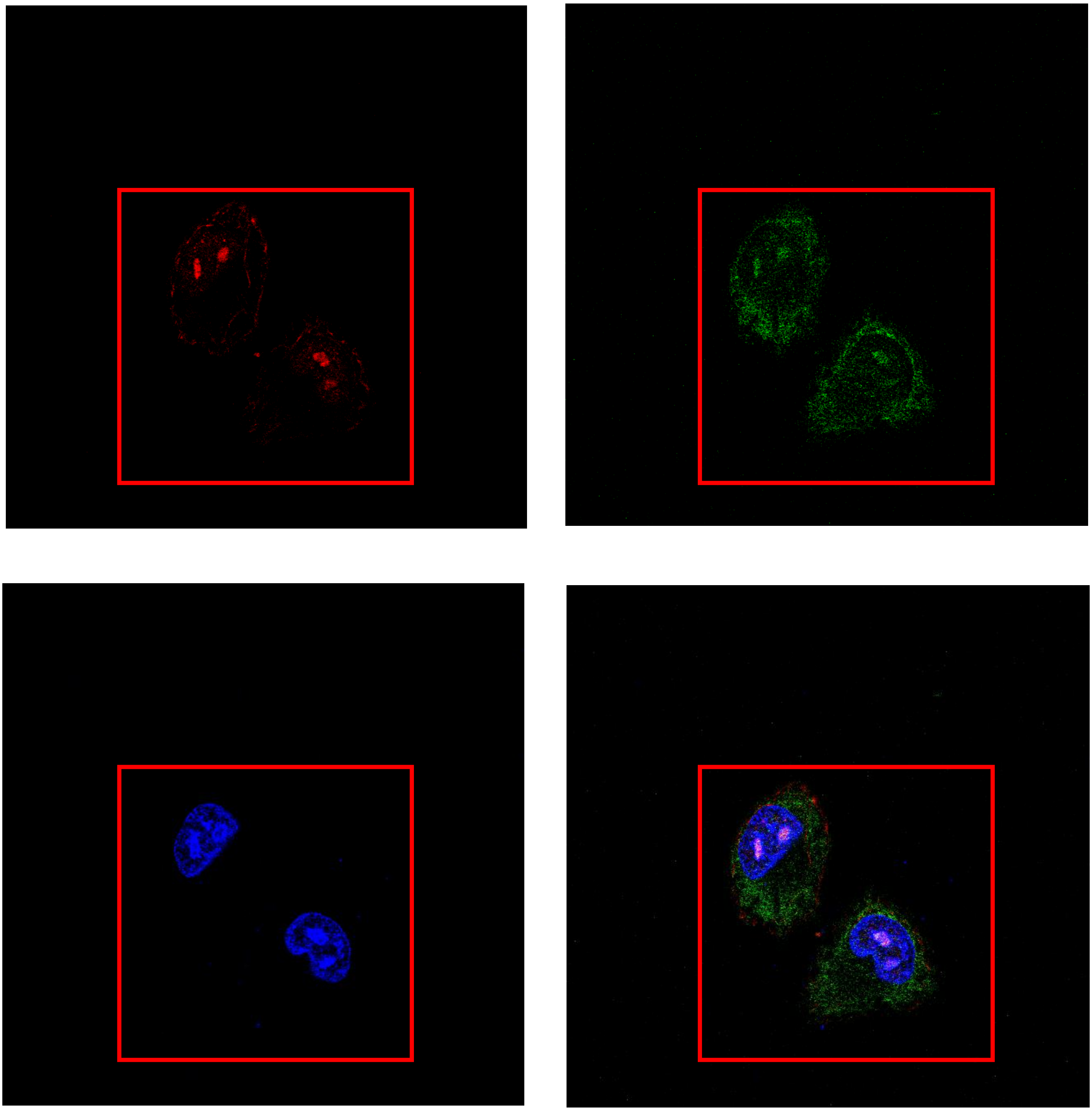

**NF90-PI**

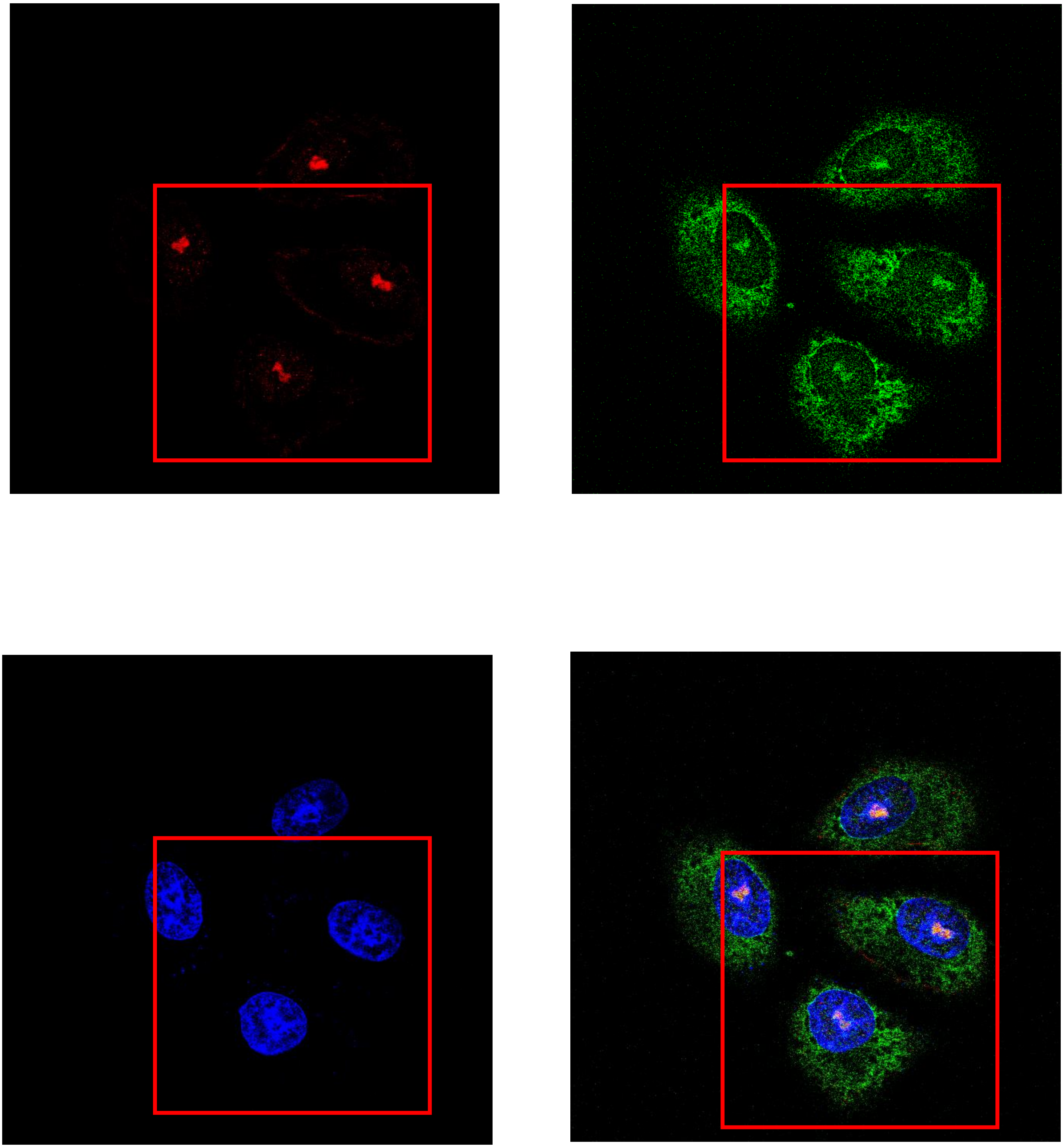

**Figure 4F**

Input:NF45

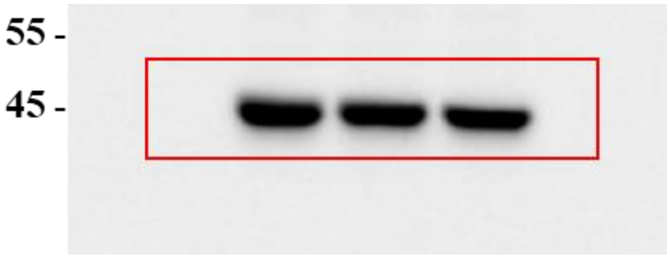


Input: NFATc1

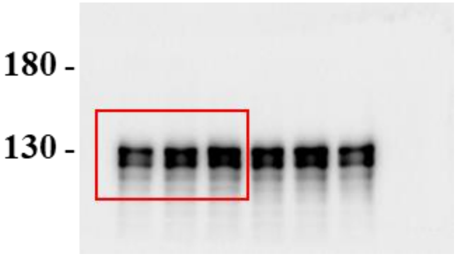


Input: NFATc2

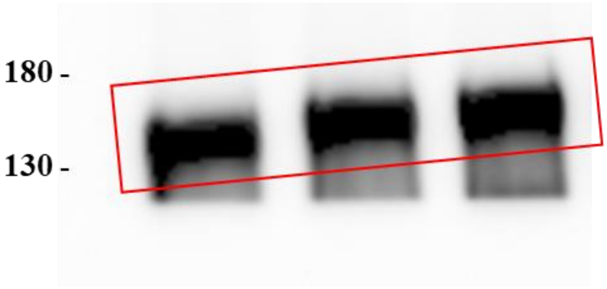


Input: GAPDH

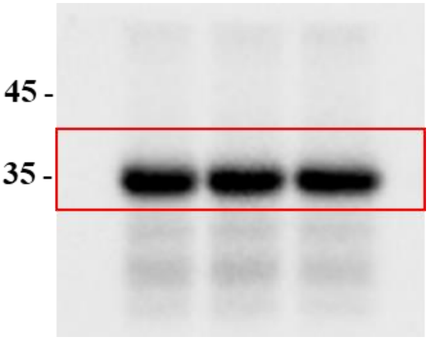


IP:NF45 IB:NF45

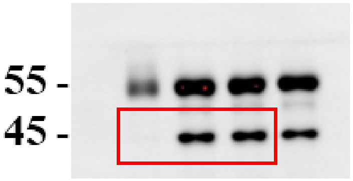


IP:NF45 IB:NFATc1

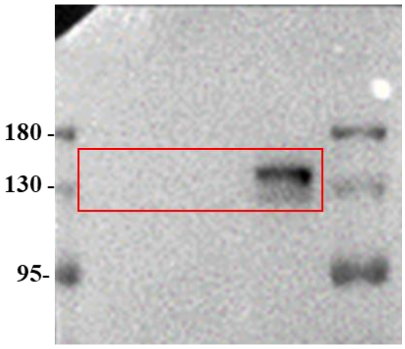


IP:NF45 IB:NFATc2

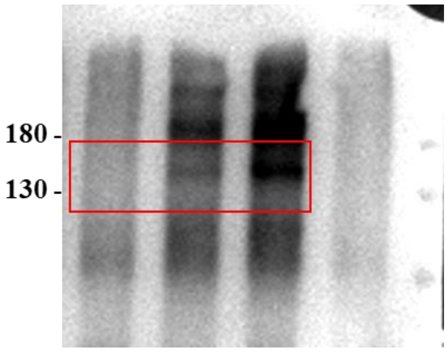

Input:NF90

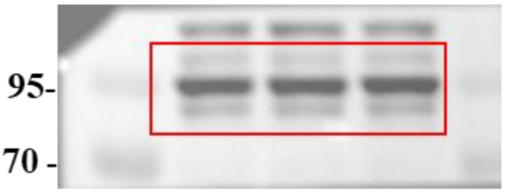


Input: NFATc1

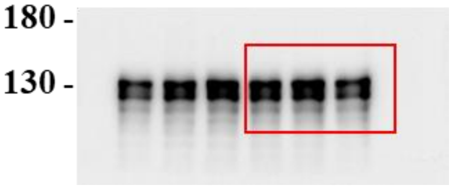


Input: NFATc2

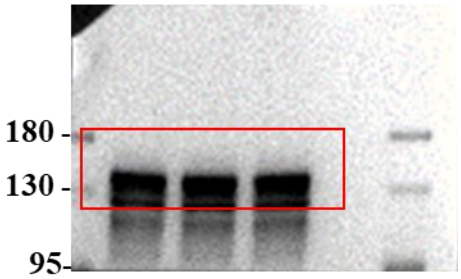


Input: GAPDH

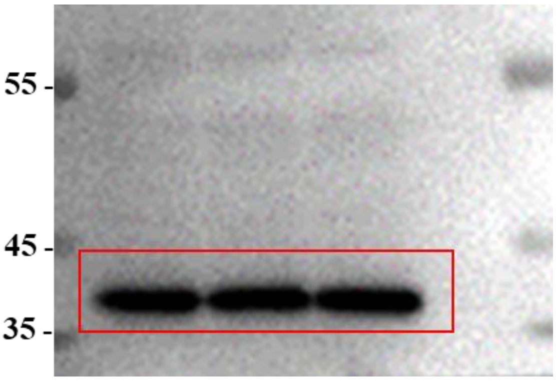


IP:NF90 IB:NF90

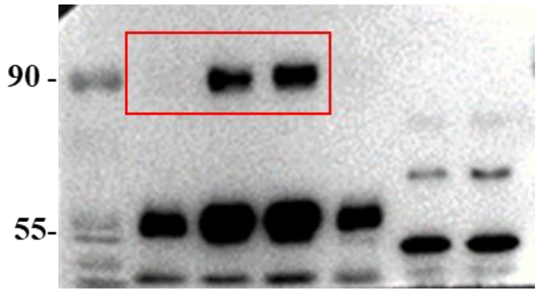


IP:NF90 IB: NFATc1

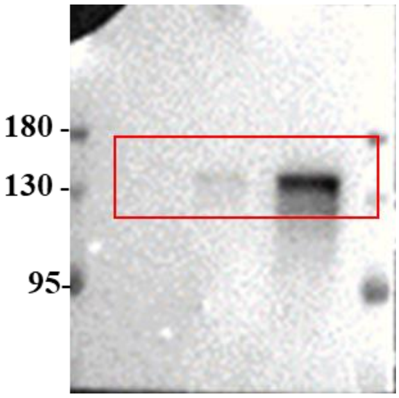


IP:NF90 IB:NFATc2

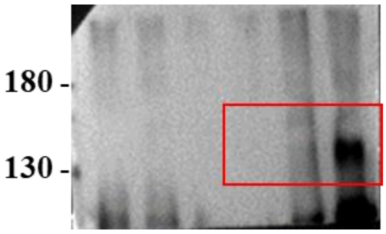

Supplement: Supplementary file 7 — Source Data for Figure 4 [file EMMM-13-e12834-s005.pdf]
